# Glucagon Enhances Chemotherapy Efficacy By Inhibition of Tumor Vessels in Colorectal Cancer

**DOI:** 10.1002/advs.202307271

**Published:** 2023-12-10

**Authors:** Yuxue Xu, Feixue Ni, Daxi Sun, Yue Peng, Yaxuan Zhao, Xiaojun Wu, Shasha Li, Xiangyu Qi, Xinkang He, Min Li, Yizi Zhou, Chao Zhang, Miao Yan, Cuifang Yao, Shuaishuai Zhu, Yang Yang, Baijiao An, Chunhua Yang, Guilong Zhang, Wenguo Jiang, Jia Mi, Xinju Chen, Pengfei Wei, Geng Tian, Yin Zhang

**Affiliations:** ^1^ School of Pharmacology Binzhou Medical University Yantai 264003 China; ^2^ Shandong Technology Innovation Center of Molecular Targeting and Intelligent Diagnosis and Treatment Yantai 264003 China; ^3^ Department of Gastroenterology Sir Run Run Shaw Hospital Zhejiang University Medical School Hangzhou 310016 China; ^4^ The First Affiliated Hospital of Henan University of Traditional Chinese Medicine No. 19 Renmin Road, Jinshui District Zhengzhou Henan 450000 China

**Keywords:** chemotherapy, colorectal cancer, glucagon, tumor angiogenesis, vascular mimicry

## Abstract

Chemotherapy is widely used to treat colorectal cancer (CRC). Despite its substantial benefits, the development of drug resistance and adverse effects remain challenging. This study aimed to elucidate a novel role of glucagon in anti‐cancer therapy. In a series of in vitro experiments, glucagon inhibited cell migration and tube formation in both endothelial and tumor cells. In vivo studies demonstrated decreased tumor blood vessels and fewer pseudo‐vessels in mice treated with glucagon. The combination of glucagon and chemotherapy exhibited enhanced tumor inhibition. Mechanistic studies demonstrated that glucagon increased the permeability of blood vessels, leading to a pronounced disruption of vessel morphology. Signaling pathway analysis identified a VEGF/VEGFR‐dependent mechanism whereby glucagon attenuated angiogenesis through its receptor. Clinical data analysis revealed a positive correlation between elevated glucagon expression and chemotherapy response. This is the first study to reveal a role for glucagon in inhibiting angiogenesis and vascular mimicry. Additionally, the delivery of glucagon‐encapsulated PEGylated liposomes to tumor‐bearing mice amplified the inhibition of angiogenesis and vascular mimicry, consequently reinforcing chemotherapy efficacy. Collectively, the findings demonstrate the role of glucagon in inhibiting tumor vessel network and suggest the potential utility of glucagon as a promising predictive marker for patients with CRC receiving chemotherapy.

## Graphical abstract

Glucagon plays an essential role in metabolism, however, its involvement in tumor development is not well understood. Here, a new biological function of glucagon is uncovered in modulation of tumor vessels. Glucagon showed a pronounced inhibition in tumor angiogenesis and vascular mimicry. This discovery suggests glucagon maybe developed as an endogenous antiangiogenic inhibitor for cancer therapy.

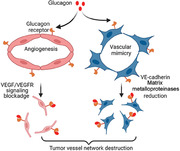

## 1 Introduction

Colorectal cancer (CRC) is a leading cause of cancer death.^[^ ^1^, ^2^ ^]^ Patients benefit considerably by combining chemotherapy with other therapies.^[^ ^3^, ^4^, ^5^, ^6^ ^]^ Fluorouracil (5‐FU), which is usually combined with other chemotherapeutics, such as the FOLFOX regimen (folinic acid, fluorouracil, and oxaliplatin), is a frequently used standard chemotherapeutic to treat patients with CRC. Although many patients with cancer benefit from 5‐FU treatment, it has several adverse effects, drug resistance, and a low response rate in some patients with cancer.^[^ ^7^ ^]^ Regardless of the cell type, 5‐FU targets rapidly replicating cells in tumors or normal tissues throughout the body, remarkably impacting non‐tumor organs.^[^ ^8^, ^9^, ^10^ ^]^ Patients receiving 5‐FU develop drug resistance after only a few cycles of therapy.^[^ ^11^ ^]^ Despite several known mechanisms of drug resistance,^[^ ^12^, ^13^, ^14^, ^15^, ^16^, ^17^ ^]^ 5‐FU‐induced drug resistance is still unclear, and the development of an effective strategy to counter 5‐FU resistance remains challenging.

The combination of 5‐FU with other anticancer agents can increase the tumor response rate and antitumor efficacy; however, ≈50% of patients do not respond to 5‐FU‐based chemotherapy.^[^ ^18^, ^19^ ^]^ Targeted therapies, such as antiangiogenic therapy, in combination with chemotherapy, significantly increase the response rate and prolong the overall survival of patients with CRC who are unresponsive to chemotherapeutics.^[^ ^20^, ^21^ ^]^ Bevacizumab is a commonly used antiangiogenic drug in combination with chemotherapeutics for CRC.^[^ ^22^ ^]^ Other antiangiogenic agents, such as aflibercept, ramucirumab, and tyrosine kinase inhibitors, have also been investigated in combination treatment, and patients with CRC benefit significantly from these antiangiogenic agents.^[^ ^21^ ^]^ However, many patients do not respond to these targeted therapies, and those who do eventually develop drug resistance. Thus, it is imperative to identify novel alternative candidates for the current antiangiogenic agents for treating patients with CRC.

Recent studies on CRC showed that glucagon (GCG) might promote tumor progression by stimulating tumor angiogenesis or tumor cell proliferation.^[^ ^23^, ^24^ ^]^ However, the underlying mechanisms are poorly understood and little is known about their role in tumor development. Glucagon, a type of polypeptide hormone physiologically secreted by pancreatic islet α cells, consists of 29 amino acids. The target organs of glucagon are mainly the liver and kidneys, owing to their high expression of glucagon receptors. Thus, it would be interesting to elucidate the role of metabolism‐related hormones, such as glucagon, in the tumor microenvironment and determine their contribution to tumor progression. Moreover, glucagon could potentially be used as, both, a target and marker for antiangiogenic based combination therapy for CRC. Thus, using in vitro and in vivo models, the study aimed to elucidate the role of glucagon. Interestingly, our data demonstrated that glucagon could inhibit vascular endothelial cell migration, proliferation, and tube formation instead of promoting endothelial cells. In two colorectal xenograft models and a patient‐derived‐xenograft (PDX) model, glucagon and 5‐FU produced notable antitumor effects. In addition, a high‐fat diet‐induced diabetic mouse tumor model confirmed that elevated glucagon levels contributed to 5‐FU efficacy enhancement. Further, glucagon could block vascular endothelial growth factor (VEGF) expression through the glucagon receptor and inhibit VEGF/VEGF receptor2 (VEGFR2) signaling, resulting in impaired tumor angiogenesis.

In addition to the primary process of sprouting angiogenesis, intussusception angiogenesis, vascular mimicry, and vessel co‐option are important for building tumor vascular/vessel networks.^[^ ^25^, ^26^, ^27^ ^]^ Many proposed mechanisms explain vascular mimicry, such as tumor microenvironment changes, hypoxia, cell‐cell junctions, and VEGF‐involved signaling, but these mechanisms have never been linked to metabolism‐related factors. In our study, we found that glucagon suppressed vascular mimicry in CRC cells, suggesting a multi‐inhibitory effect of glucagon on the tumor vessel network.

Moreover, glucagon‐encapsulated PEGylated liposomes with tumor‐targeting folic acid molecules (GCG@LFA) were constructed and showed increased therapeutic efficiency in a tumor model, suggesting glucagon as a potential agent for adjuvant treatment. In summary, we demonstrated that glucagon can inhibit colorectal tumors by disrupting angiogenesis and vascular mimicry, which may be translated into clinical practice for further validation.

## 2 Results

### 2.1 High Glucagon Expression is Correlated with Improved Survival of Patients with CRC

**Figure 1:**
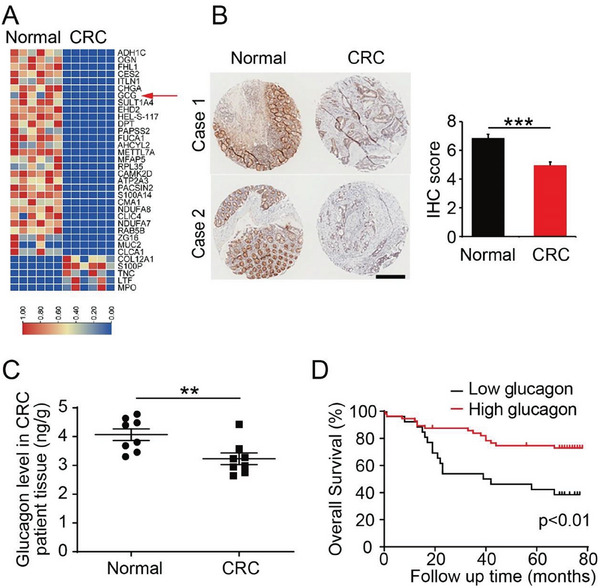
Low glucagon expression in colorectal tumor tissue. A) Heatmap of differentially expressed proteins in normal and tumor tissues from patients with colorectal cancer (CRC, *n* = 6). GCG, glucagon. B) Immunohistochemical (IHC) staining of glucagon in clinical samples. The relative IHC scores (right panel) were quantified from the corresponding staining images. *n* = 82. Scale bar: 400 µm. C) enzyme‐linked immunosorbent assay detection of glucagon concentration from patient samples (*n* = 6). D) Correlation of glucagon expression and overall survival in patients with CRC (*n* = 82).

To clarify the role of glucagon and its clinical relevance in CRC, we compared glucagon expression levels in tumor and normal tissues in 82 clinical samples of patients with CRC. Interestingly, glucagon was highly expressed in majority of the normal tissue samples (92.7%) but not in tumor tissues (68.3%) (Table S1, Supporting Information). In advanced disease stage, the tumor showed lower expression than that at early stages of the disease (Table S2, Supporting Information). Proteomic analysis of samples from five patients with CRC revealed that glucagon expression decreased considerably in colorectal tumors compared to that in normal tissues (**Figure** **1**A). Immunohistochemical staining further demonstrated remarkably lower glucagon levels in tumors than that in normal tissues (Figure 1B). Since glucagon is mainly produced in the pancreatic islets and have functions in liver, it is surprising that glucagon levels are altered in colorectal tissues. To confirm glucagon expression in CRC samples, we stained pancreatic islets from patients with pancreatic cancer as a positive control to test if the staining worked correctly. The results showed a clear positive signal in pancreatic islets, but, interestingly, glucagon levels significantly decreased in pancreatic tumor tissues, indicating that decreased glucagon levels might be a general phenomenon in tumor tissues (Figure S1A, Supporting Information). To further validate glucagon expression, enzyme‐linked immunosorbent assay (ELISA) was performed to measure the glucagon levels in samples of patients with CRC. Consistent with the staining results, ELISA data revealed that glucagon levels decreased significantly in the tumor tissue (Figure 1C).

Next, we analyzed clinical samples from 82 patients with CRC to determine whether glucagon expression levels correlated with patient survival. Surprisingly, high glucagon levels were associated with improved survival, whereas low glucagon levels significantly decreased survival (Figure 1D). In addition, data from the cancer genome atlas program (TCGA) showed a 5‐year survival rate of 74% for patients with high glucagon levels, compared to only 47% for those with low glucagon expression (Figure S1B, Supporting Information). These data suggest that glucagon may contribute to the improved survival of patients with CRC.

### 2.2 Glucagon Inhibited Tumor Blood Vessels

**Figure 2:**
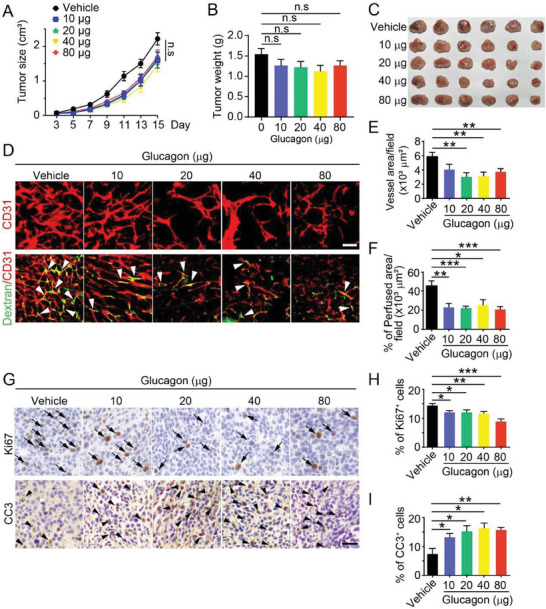
Glucagon inhibits tumor blood vessels. A) Tumor growth curves after treatment with different glucagon doses (*n* = 9–10 mice per group). The experiment was repeated twice. B) Quantification of CT26 tumor weight. *n* = 9–10 mice per group. C) CT26 tumor images of different treatment groups. D) Whole mount staining with CD31 antibodies for CT26 tumor blood vessels (upper panel) and perfusion of dextran 2000 kDa (lower panel). The white arrow indicates perfused vessel structure. Scale bar: 50 µm. E) Quantification of CD31^+^ area in glucagon‐treated tumor tissues using Adobe Photoshop 2022 software (*n* = 8). F) Quantification of the percent perfused vessel area of dextran 2000 kDa after glucagon treatment. Mean ± standard error of mean (SEM, *n* = 8). G) IHC analysis of proliferation and apoptosis using Ki67 or cleaved caspase‐3 (CC3) staining of CT26 tumor tissue paraffin sections. Arrows indicate proliferating cells and arrowheads indicate apoptotic cells. Scale bar: 20 µm. H) Quantification of Ki67^+^ cell percentages in CT26 tumor tissues. Mean ± SEM, *n* = 5;I) Quantification of CC3^+^ cells in CT26 tumor tissues. Mean ± SEM, *n* = 5.

To investigate the role of glucagon in CRC and its mechanism of action in improving survival, glucagon was administered to a mouse CRC tumor model. CT26, a mouse CRC cell line, was used to establish a xenograft tumor model in BALB/c mice. Different doses of glucagon were injected intraperitoneally into tumor‐bearing mice. Although tumor growth and weight did not show statistically significant differences (**Figure** **2**A–C), glucagon presented an inhibitory trend on tumor size and weight. Thus, glucagon treatment alone may not be as powerful as blocking tumor growth, but might produce a synergistic antitumor effect when combined with other cancer therapies, since patients usually receive several anticancer treatments.

Glucagon is suggested to play a role in angiogenesis; therefore, we examined tumor vessels after glucagon treatment. Interestingly, blood vessels were significantly decreased by glucagon treatment, suggesting the vessel inhibition ability of glucagon (Figure 2D,E). In addition, functional analysis of the blood vessels was performed using a perfusion assay with a fluorescence‐labeled dextran of 2000 kDa. Dextran revealed that vessel perfusion decreased after glucagon treatment (Figure 2D,F). Thus, glucagon may impair tumor vessel function, reflecting its antiangiogenic effect. Interestingly, glucagon treatment inhibited cell proliferation (indicated by decreased Ki67 levels) and induced apoptosis (indicated by increased cleaved caspase‐3 levels) in the tumor tissues (Figure 2G,H,I).

### 2.3 Glucagon Sensitized Chemotherapy on CRC Tumor

**Figure 3:**
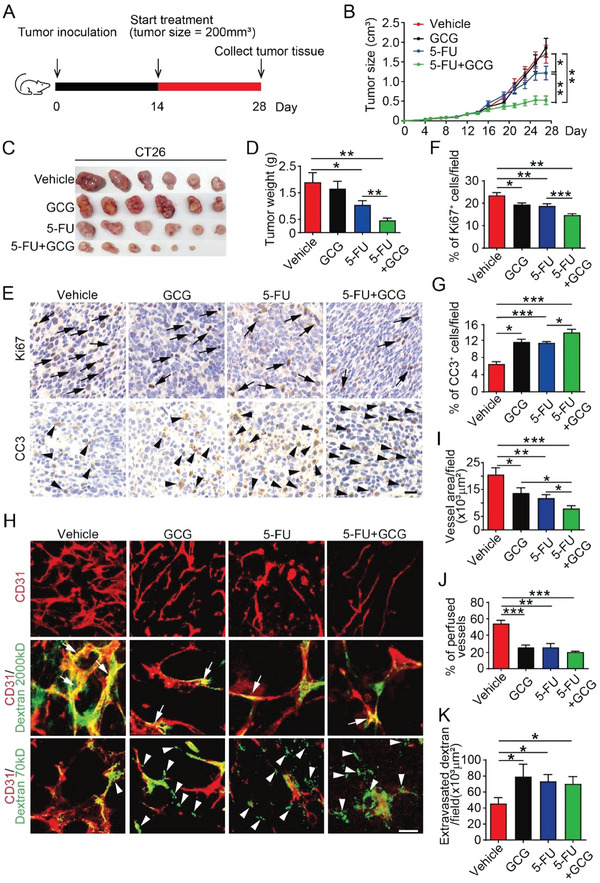
Glucagon sensitizes 5‐FU induced antitumor effect. A) Schematic representation of the treatment regimen. Treatment with vehicle, 5‐FU, glucagon, or 5‐FU+glucagon was initiated two weeks after CT26 tumor cell inoculation when the average tumor size reached ≈200 mm^3^. After 2‐week treatment, tumor tissues were harvested and analyzed accordingly. (B) Growth curves of CT26 tumors under different treatments. Dose: GCG, 20 µg; 5‐FU, 25 mg kg^−1^. Vehicle:Saline. *n* = 9–10 mice per group. C) CT26 tumor images and D) tumor weights in different treatment groups. (E) Immunohistochemical analysis of CT26 tumor proliferation and apoptosis. Arrows indicate proliferative cells and arrowheads indicate apoptotic cells. Scale bar: 20 µm. F) Quantification of Ki67 positive cells in different treatment groups (*n* = 6). G) Quantification of CC3 positive cells in different treatment groups (*n* = 6). H) Images of CT26 tumor blood vessels perfused with dextran 2000 kDa, and dextran 70 kDa. The blood vessels are red and dextran is green. Arrows indicate perfused vessels, and arrowheads indicate leaked dextran 70 kDa. Bar: 20µm. I) Quantification of blood vessel area (*n* = 6). J) Quantification of perfused vessels (*n* = 6). K) Quantification of extravasated dextran (*n* = 6).

As glucagon alone did not significantly inhibit tumor growth, we hypothesized that the improved survival of patients with CRC might be due to the combined effect of glucagon with other anticancer treatments. Chemotherapy is a standard treatment for patients with CRC, which is usually combined with other targeted drugs, such as antiangiogenic agents. These agents, including bevacizumab, in combination with chemotherapy, are usually administered to patients with metastatic or late‐stage CRC.^[^ ^29^ ^]^ However, bevacizumab alone is clinically inefficient, although it is highly effective preclinically in blocking tumor growth and metastasis.^[^ ^30^, ^31^ ^]^ Owing to its antiangiogenic potential, the combination of glucagon and chemotherapeutic drugs may produce a synergistic antitumor effect, similar to the combinatory use of bevacizumab and chemotherapeutic drugs in clinical practice. To validate this hypothesis, we chose 5‐FU, a standard chemotherapeutic agent used to treat CRC, in combination with glucagon in our xenograft model. Treatment was initiated when the tumor size reached ≈200 mm^3^ (**Figure** **3**A). After treatment for two weeks, the combination treatment group showed the greatest antitumor effect. The tumor size was 60% lesser in the combination group than that in the vehicle‐treated group, whereas low‐dose glucagon treatment alone did not show any inhibitory effect (Figure 3B,C). Quantification of tumor weight also showed that tumor growth was further inhibited by combination treatment compared to that with 5‐FU treatment (Figure 3D). In addition, combination treatment further decreased the number of proliferative cells and induced apoptosis in the tumor tissues (Figure 3E–G). Along with increased apoptosis, tumor hypoxia was also increased in the glucagon‐treated group and further increased in the combination treatment group (Figure S2A, Supporting Information).

To confirm our hypothesis, we established a glucagon overexpressed tumor cell line, OE‐GCG‐CT26, to test whether endogenous glucagon would show the same antitumor effect as 5‐FU (Figure S2B, Supporting Information). As expected, glucagon overexpression significantly reduced tumor growth when combined with 5‐FU treatment, although overexpressed glucagon alone did not induce significant tumor blockade (Figure S2C–E, Supporting Information). Collectively, these data demonstrate that chemotherapy combined with glucagon produces a synergistic antitumor effect.

### 2.4 Glucagon Impaired Tumor Vessel Structure and Function

Furthermore, we analyzed the structure and function of tumor blood vessels to investigate the mechanism underlying the enhance antitumor effect of combination therapy. As observed earlier, blood vessel density was decreased by glucagon treatment alone, and the combination of GCG+5‐FU further decreased vessel density (Figure 3H,I). In the vessel perfusion assay, dextran 2,000 kDa decreased in the combination therapy group (Figure 3H, J), indicating a perfusion defect. Similarly, the 70 kDa dextran, which indicates vessel permeability, was further increased outside the blood vessels by combination treatment (Figure 3H–K). Transmission electron microscopy was used to examine the leaky vessels and endothelial cells under these treatments. Analysis of tumor vascular endothelial cells revealed a disorganized and torched cell membrane structure (Figure S3A, Supporting Information). In addition, a significant decrease in VE‐cadherin, a key cell‐cell junction protein in endothelial cells, was found in the combination treatment group, revealing a loosened junction between the endothelial cells, resulting in high permeability (Figure S3B,C, Supporting Information). Thus, glucagon not only impaired tumor angiogenesis but also blocked vessel perfusion and increased permeability, which blocked blood supply to the tumor tissue. Thus, glucagon enhances the antitumor effect of 5‐FU by destroying the tumor vessels.

### 2.5 Glucagon Inhibits Angiogenesis and Vascular Mimicry

**Figure 4:**
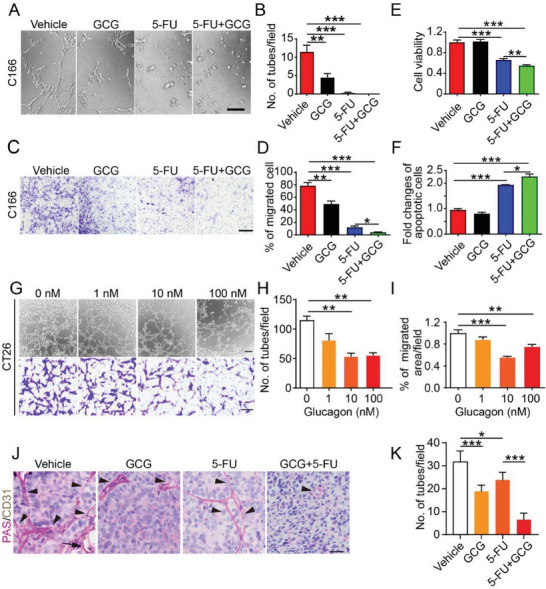
Glucagon inhibits angiogenesis and vascular mimicry. A) Tube formation assay of endothelial cells (C166 cells) treated with control, glucagon (GCG, 10 nm), 5‐FU (100 ng mL^−1^), or 5‐FU+GCG. Bar: 50 µm. The experiment was repeated three times. B) Quantification of tube formation (*n* = 3). C) Migration assay of different treatments on endothelial cell (C166 cells). Bar: 200 µm. The experiment was repeated twice. GCG (10 nm), 5‐FU (100 ng mL^−1^). D) Quantification of migrated endothelial cells on different treatments (*n* = 3). (E) Quantification of endothelial cell viability on different treatments (*n* = 3). F) Quantification of endothelial cell apoptosis on different treatments (*n* = 3). G) Tube formation and migration assays on tumor cells (CT26 cells). Bar: 100 µm. All experiments were repeated three times. H) Quantification of tube numbers in CT26 cells (*n* = 3). I) Quantification of migrated CT26 cells (*n* = 3). J) Staining of CT26 tumor cells forming vessels. Arrowheads indicate tumor cells forming pseudo vessels and arrows indicate PAS/CD31 double‐stained blood vessels. (K) Quantification of pseudo vessels in different treatment groups (*n* = 6).

Glucagon inhibited tumor blood vessels; however, whether this endothelial cell inhibition occurs in a direct manner warrants further validation. As glucagon plays a role in elevating glucose levels, which may impact tumor growth, we measured the glucose levels in our mouse model. Surprisingly, the glucose levels in all groups showed no differences (Figure S4A, Supporting Information), and glucagon‐treated mice did not show elevated glucose levels, which indicated that the glucagon‐induced synergistic antitumor effect might directly affect endothelial and tumor cells. Therefore, we treated endothelial cells with different doses of glucagon and analyzed cell proliferation and apoptosis. The CCK8 assay indicated dose‐dependent endothelial cell inhibition by glucagon, while flow cytometry analysis also demonstrated an increased apoptotic cell population following glucagon treatment (Figure S4B,C, Supporting Information). We observed that glucagon significantly blocked endothelial cell tube formation in vitro, and combination treatment showed an even more pronounced effect. The combination treatment blocked almost all endothelial cell tubes, while glucagon or 5‐FU treatment alone still formed a few tubes or cell‐cell contacts (**Figure** **4**A,B). The migratory ability of endothelial cells is key for angiogenesis; however, glucagon‐treated endothelial cells partially lost their mobility in the Transwell assay. When combined with 5‐FU treatment, this anti‐migration effect was more significant (Figure 4C,D). Moreover, combination therapy further decreased endothelial cell proliferation and increased apoptosis (Figure 4E,F).

Vascular mimicry is critical in tumor development. Tumor cells can build vessel‐like structures that connect to the vascular network. These pseudo‐vessels can improve blood supply and contribute to tumor growth and metastasis.^[^ ^32^, ^33^ ^]^ Therefore, we examined the vascular mimicry parameters of CT26 cells, both in vitro and in vivo. In the tube formation assay, CT26 cells formed tubes, which were inhibited by glucagon dose‐dependently (Figure 4G,H). Interestingly, CT26 cell migration was also blocked by glucagon (Figure 4G–I). However, challenging both, mouse and human CRC cells with glucagon did not alter cell viability, indicating that glucagon had no effect on tumor cell proliferation (Figure S4D,E, Supporting Information).

In vivo, we double‐stained the endothelial marker, CD31, and the vessel basement membrane using Periodic Acid‐Schiff (PAS) staining, allowing us to distinguish real blood vessels (CD31^+^/PAS^+^) and pseudo vessels (CD31^−^/PAS^+^). Interestingly, the peripheral tumor tissues were highly vascularized, as indicated by intensive CD31^+^/PAS^+^ double‐positive vessels, whereas in the intra‐tumoral area, vascular mimicry was dominated by strongly stained CD31^−^/PAS^+^ vessels (Figure S4F, Supporting Information). Consistent with the cell model, glucagon significantly reduced the mimicry vessels and further reduced the pseudo‐vessels when combined with 5‐FU (Figure 4J–K). Matrix metalloproteinases and VE‐cadherin are markers associated with vascular mimicry in tumors.^[^ ^34^, ^35^, ^36^ ^]^ Thus, we tested these markers in glucagon‐treated tumors. Upon glucagon treatment, MMP‐2, MMP‐9, and VE‐cadherin levels decreased, suggesting decreased vascular mimicry (Figure S4G,H, Supporting Information). Thus, glucagon inhibited vascular mimicry, which contributed to tumor suppression when combined with 5‐FU. Collectively, both in vitro and in vivo studies demonstrated that glucagon inhibited tumor angiogenesis and vascular mimicry. The combination of glucagon and 5‐FU further decreased blood vessel and pseudo‐vessel density, which contributed to tumor growth inhibition.

### 2.6 Glucagon Inhibited VEGF‐Dependent Tumor Angiogenesis through Glucagon Receptor Signaling

**Figure 5:**
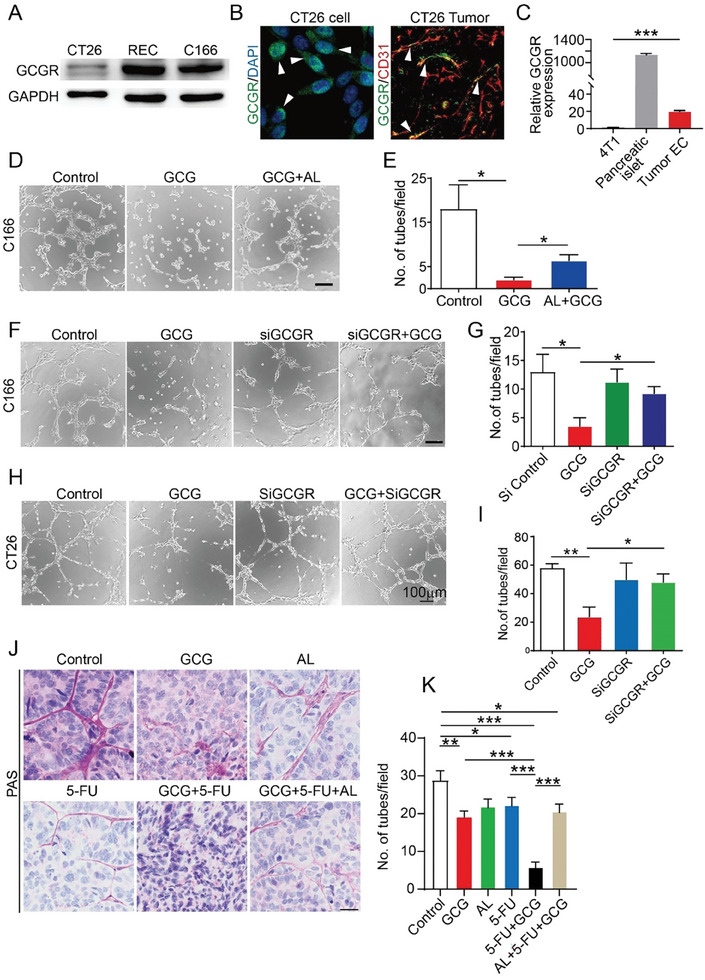
Glucagon‐induced endothelial cell and vascular mimicry inhibition are reversible by glucagon receptor blockade or knock down. A) Glucagon receptor expression in, both, the tumor and endothelial cells was determined using western blotting. REC: Retinal endothelial cell. The experiment was repeated three times. B) Staining of glucagon receptor (GCGR) in CT26 cells and tumor tissues. C) GCGR mRNA expression in tumor endothelial cells. The breast cancer cell line 4T1 was used as a negative control and mouse pancreatic islets were used as a positive control (*n* = 3). D) Tube Formation in C166 cells. Bar: 50 µm. The experiment was repeated three times. GCG, 10 nm; AL, 0.5 µm. E) Quantification of tube formation (n = 3). F) Tube formation assay results for GCGR‐knockdown C166 cells. Bar: 50 µm. The experiment was repeated twice. GCG, 10 nm; siGCGR, 10 µm. G) Quantification of tube formation for GCGR‐knockdown (n = 3). H) Tube formation of CT26 cells after GCGR‐knockdown (*n* = 3). I) Quantification of tubes (*n* = 3). J) Periodic Acid‐Schiff staining of CT26 tumor cell formed pseudo vessels. Bar = 100 µm. (K) Quantification of mimicry vessels using different treatments (*n* = 5–8).

Glucagon aggravated endothelial cell dysfunction following 5‐FU treatment; however, the underlying mechanism was unclear. We hypothesized that glucagon disrupts angiogenic signaling through its receptors and downstream pathways. Therefore, expression of the glucagon receptor (GCGR) was examined. In both endothelial and tumor cells, western blot analysis showed that GCGR were remarkably expressed in these cells (**Figure** **5**A). Furthermore, immunofluorescent staining confirmed that the GCGR was expressed on CT26 and endothelial cells in the tumor tissue (Figure 5B). We also isolated endothelial cells from CT26 tumor tissues by magnetic‐activated cell sorting, followed by quantitative polymerase chain reaction using mouse GCGR primers. The results showed significant expression of GCGR mRNA in endothelial cells compared to that in the negative control of 4T1 cells (Figure 5C). These data confirmed the expression of GCGR in endothelial cells. To validate whether glucagon induces antiangiogenic effects and enhances 5‐FU efficacy via its receptor, we introduced a glucagon pathway inhibitor, LY2409021, also known as adomeglivant (AL), as a GCGR antagonist. A phase II trial by Eli Lily demonstrated that AL can block glucagon signaling in type 2 diabetes, resulting in reduced glucose levels.^[^ ^37^, ^38^ ^]^ As shown in Figure 5D,E, glucagon blocked endothelial cell tube formation; however, AL rescued this phenotype. Similarly, the knockdown of GCGR in endothelial cells using small interfering (si)RNA restored tube formation (Figure 5F,G).

In the in vivo tumor model, we established a stable glucagon receptor‐knockout CT26 cell line to test its function in tumors (Figure S5A, Supporting Information). GCGR‐KD tumors showed no changes in tumor growth rate or weight; however, the tumor was not further inhibited on treatment with 5‐FU plus glucagon, compared to 5‐FU treatment alone. This GCGR‐deficient tumor (red curve) regained 5‐FU resistance relative to control tumor treated with 5‐FU plus glucagon (blue curve) (Figure S5B–D, Supporting Information). Thus, glucagon induced anti‐vessel effects directly through the GCGR.

VEGF is one of the main driving forces of endothelial cell migration, proliferation, and tube formation. To test whether glucagon‐induced endothelial cell tumor formation was VEGF‐dependent, we challenged endothelial cells with different combinations of VEGF and glucagon. Interestingly, VEGF largely rescued C166 cells from tube inhibition, and glucagon‐induced inhibition was almost at the same level as that induced by an anti‐VEGF antibody (Figure S5E,F, Supporting Information). Thus, endothelial cell inhibition by glucagon was VEGF‐dependent. We also examined key molecules in the VEGF/VEGFR signaling pathway, which are critical for endothelial cell activation. Immunoblotting showed that glucagon decreased, both, VEGFR1 and VEGFR2 phosphorylation, resulting in decreased activities of AKT and ERK, which are key downstream signaling proteins which control endothelial cell migration and survival (Figure S5G, Supporting Information). Interestingly, 5‐FU produced a similar effect by inhibiting signal activation, with the combination treatment producing a more significant blockage. Additionally, VE‐cadherin expression reduced by 5‐FU+GCG treatment (Figure S5G, Supporting Information). Collectively, these data demonstrate that glucagon inhibits angiogenesis by directly targeting endothelial cells via the conventional VEGF/VEGFR signaling axis.

### 2.7 Glucagon‐Induced Vascular Mimicry Inhibition was Reversed by Blocking GCGR

To validate whether the glucagon‐induced vascular mimicry defect was also induced by GCGR, we knocked down GCGR expression in CT26 cells. In the tube formation assay, siGCGR largely recovered CT26 cells from forming tubes (Figure 5H‐I). In vivo, AL was administered alone or in combination with 5‐FU+glucagon to tumor‐bearing mice. PAS staining of the tumor sections revealed that the AL did not reduce vascular mimicry, but could restore pseudo‐vessel density after 5‐FU+GCG treatment (Figure 5J‐K). Similarly, AL did not change tumor growth or angiogenesis, but reversed the glucagon produced by a synergistic effect on the inhibition of tumor angiogenesis, thus recovering tumor growth or vessels (Figure S6A–D, Supporting Information). Similarly, AL restored tumor vessel perfusion and decreased permeability, which promoted tumor growth (Figure S6E–I, Supporting Information). These data validate our hypothesis that glucagon enhances 5‐FU‐suppression of tumor growth via the glucagon receptor.

### 2.8 Treatment with 5‐FU Showed Improved Sensitivity in a Diabetic Mouse Tumor Model

Many patients with diabetes have elevated levels of circulating glucagon. However, the endogenous glucagon contributing to tumor growth needs to be validated. To identify any potential clinical correlation between glucagon levels and tumor growth, a diabetic mouse model was established after 12 weeks of high‐fat diet (HFD) feeding, with increase in serum glucagon levels (Figure S7A, Supporting Information). Interestingly, the HFD+5‐FU group showed much stronger tumor suppression than that of the normal chow diet (NCD+5‐FU) group, whereas there were no differences in tumor growth and weight between the HFD and NCD groups (Figure S7B–D, Supporting Information). Tumor angiogenesis was inhibited in the HFD group, blood vessels were further reduced in HFD+5‐FU tumors compared to those in the NCD+5‐FU group (Figure S7E,F, Supporting Information). Similarly, decreased perfusion and increased extravasation of tumor vessels were detected in HFD tumors, similar to the exogenous glucagon treatment, indicating that elevated endogenous glucagon levels might contribute to the antiangiogenic effect (Figure S7F–H, Supporting Information). These data corroborated the findings from non‐diabetic tumor models with glucagon treatment, which further raises the possibility that endogenous glucagon derived from HDF models may contribute to sensitizing 5‐FU treatment on CRCs.

### 2.9 Glucagon Produced Synergistic Antitumor Effects in Clinically Relevant Tumor Models and Clinical Data Set Analysis

**Figure 6:**
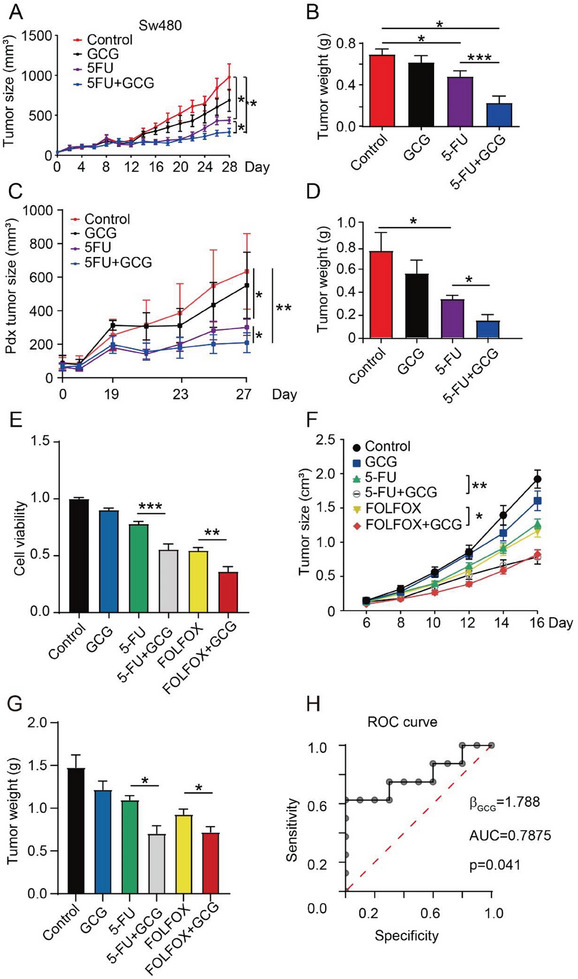
Clinical relevance study of glucagon in improving chemotherapy. A) Tumor growth curve of SW480 cells (Control group, *n* = 7; GCG, 5‐FU, and 5‐FU+GCG groups, *n* = 8), GCG, 20 µg per mouse; 5‐FU, 25 mg kg^−1^. B) Tumor weight of SW480 (*n* = 6). C) Growth curves of CRC PDX tumors after different treatments. GCG, 20 µg per mouse; 5‐FU, 25 mg kg^−1^ (*n* = 4). D) Quantification of CRC tumor weight (n = 4). E) CT26 cell viability after different treatments. GCG, 10 nm; 5‐FU, 50 ng mL^−1^; Oxaliplatin, 5 µm; Calcium Folinate, 12.5 µm (*n* = 3). F) Tumor growth of CT26 tumors under different treatment conditions (*n* = 6). G) Quantification of CT26 tumor weight under different treatment conditions (*n* = 6). H) The correlation between glucagon expression levels and chemotherapy sensitivity using logistic regression analysis is presented in the form of a receiver operating characteristic curve (*n* = 18 male patients in the late stage of the disease).

We tested our hypotheses using several clinically relevant models to translate our findings into clinical practice. First, we established a human colorectal tumor model using SW480 cells. Similar to the mouse tumor model, glucagon significantly enhanced 5‐FU therapy for tumor growth suppression (**Figure** **6**A,B; Figure S8A, Supporting Information). To confirm our findings, we used a PDX tumor model with CRC patient‐derived tumor tissues. Consistently, the PDX model also demonstrated that combination treatment could better inhibit tumor growth (Figure 6C,D). Moreover, staining of blood vessels with CD31 demonstrated the inhibitory effect of glucagon on tumor angiogenesis (Figure S8B, Supporting Information).

Since 5‐FU is seldom used as a monotherapy in cancer treatment for patients with CRC, we applied a commonly used clinical regimen of chemotherapy, FOLFOX (folinic acid, fluorouracil, and oxaliplatin), in our tumor model to generalize our findings. Interestingly, combining 5‐FU or FOLFOX with glucagon treatment significantly decreased tumor cell viability (Figure 6E). Consistent with previous data, glucagon further improved FOLFOX treatment in terms of tumor growth inhibition, as it did when combined with 5‐FU (Figure 6F,G; Figure S8C, Supporting Information).

To further investigate the clinical correlation between glucagon expression and chemotherapy response, we analyzed clinical data from the GEO dataset GSE69657 using logistic regression. Patients with CRC at a similar tumor stage who were treated with chemotherapy were included. The outcome was recorded as non‐responder or responder, and glucagon expression was determined using RNA‐seq. The odds ratio (β_GCG_) was positive, indicating that an increase in glucagon positively correlated with the outcome of the responder (Figure 6H). In addition, the receiver operating characteristic curve also showed an area under the curve value close to 1, with a p‐value less than 0.05, which suggests that the glucagon level may be used as an accurate model to predict chemotherapy response in patients with CRC.

### 2.10 Liposomal Delivery of Glucagon Significantly Improved 5‐FU‐Mediated Tumor Suppression

**Figure 7:**
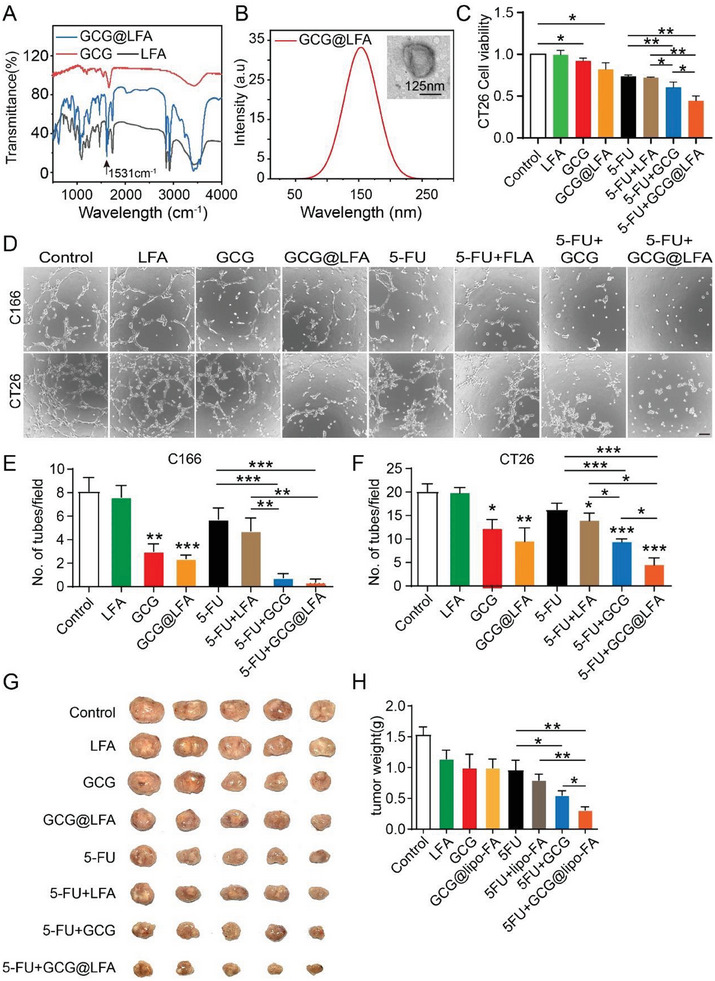
Liposomal delivery of glucagon significantly improved 5‐FU‐induced tumor suppression. A) The Fourier transform infrared spectra of GCG, LFA, and GCG@LFA. B) Size distribution of GCG@LFA. The inset in the top‐right image shows the morphology of GCG@LFA, as observed using transmission electron microscopy. C) Cell viability analysis of CT26 cells exposed to different treatments for 48 h using the CCK‐8 assay. GCG: 10 nm; 5‐FU, 50 ng mL^−1^ (*n* = 3). D) Tube formation in C166 and CT26 cells exposed to different treatments for 48 h GCG: 10 nm. Doses used in the other treatment groups were equivalent to those used in the glucagon treatment group (*n* = 3). E) Quantification of tubes for C166 cells based on the experiment in Figure 6D (*n* = 3). F) Quantification of tubes for CT26 cells based on the experiment in Figure 6D (*n* = 3). G) Images of CT26 tumors subjected to different treatments. Dosing: GCG, 20 µg; GCG@LFA, 20 µg; 5‐FU, 12.5 mg kg^−1^. H) Quantification of tumor weight (*n* = 5–8).

As illustrated above, glucagon can inhibit tumor angiogenesis and vascular mimicry, and possesses chemosensitization capabilities. The delivery of glucagon to patients with cancer may be a potential approach to increase chemotherapy efficacy. Clinically, glucagon injections are primarily used to treat hypoglycemia, cardiogenic shock, and other adverse reactions, including nausea, vomiting, hyperglycemia, and hypokalemia. Based on the defects of directly delivering glucagon, improving the therapeutic efficacy and safety of glucagon by targeted delivery to tumor locations and selective accumulation is critical for its in vivo application. Liposomes, which are nanosized phospholipid bubbles, have received considerbale attention as potential drug carriers owing to their easy preparation and surface engineering, high biocompatibility, and drug‐loading capacity.^[^ ^34^ ^]^ Moreover, surface PEGylation and the modification of liposomes with targeting ligands (such as hyaluronic and folic acid) have made liposomal delivery platforms one of the most promising drug carriers for CRC treatment.^[^ ^35^ ^]^ In this study, to increase the tumor suppression efficiency of glucagon in combination with 5‐FU, folic acid (FA)‐conjugated and PEGylated liposomes (LFA) were used for glucagon delivery. The synthesized FA‐conjugated PEGylated liposomes carrying glucagon (GCG@LFA; Figure S9A, Supporting Information) were systematically characterized. As shown in **Figure** **7**A, an emerging characteristic peak (1531 cm^−1^) of GCG@LFA was observed, demonstrating that GCG had been embedded inside LFA. In addition, size distribution of the GCG@LFA particles was analyzed using dynamic light scattering, with an average particle size of 152.7 nm (Figure 7B). The encapsulation efficiency of glucagon into LFA was 80.33%, as determined using ELISA. Furthermore, an in vitro study was performed to verify the tumor‐targeting capacity of GCG@LFA and to enhance its tumor inhibitory efficiency. FITC‐ and glucagon‐co‐loaded LFA nanosystems were constructed and demonstrated obvious CT26 cell membrane recognition and uptake, which could be significantly blocked by adding free folic acid (Figure S9B, Supporting Information). We tested whether GCG@LFA could further increase the antitumor effect. CT26 cells were treated with glucagon, GCG@LFA, or 5‐FU. Cell viability assay revealed that the number of tumor cells decreased on combining 5‐FU with GCG@LFA compared to those with GCG+5‐FU, although no significant changes were observed with GCG@LFA compared to those with glucagon treatment alone (Figure 7C). Functional analysis of, both, endothelial and tumor cells indicated that GCG@LFA+5‐FU completely inhibited tube formation by tumor or endothelial cells, whereas in the GCG+5‐FU group, only a few tubes remained (Figure 7D–F). This suggests that GCG@LFA can increase tumor inhibition in combination with 5‐FU. In addition, folic acid (FA)‐conjugated and PEGylated liposomes encapsulating indocyanine green (ICG) and glucagon (ICG/GCG@LFA) were prepared for in vivo near‐infrared (NIR) imaging of BALB/c mice using an optical in vivo imaging system. Both the fluorescence emission spectra and corresponding fluorescence images of GCG@LFA and ICG/GCG@LFA at equivalent ICG concentrations demonstrated successful encapsulation of ICG in the liposomal system (Figure S9C, Supporting Information). The fluorescence emission spectrum exhibited an emission wavelength of ≈820 nm (Figure S9D, Supporting Information). Real‐time NIR imaging of mice bearing CT26 cell transplantation tumors was performed using a fluorescence imaging system after tail vein injection of ICG/GCG @LFA. As shown in Figure S9E,F (Supporting Information), ICG/GCG/FLA was successfully delivered to the tumor tissues after 3 h and reached the maximum intensity at 24‐h, following which the signal gradually faded after 48 h. Accordingly, we performed in vivo experiments using the CT26 tumor model and found that GCG@LFA+5‐FU resulted in significant tumor suppression. The inhibition reached 70%, and compared to GCG+5‐FU, GCG@LFA+5‐FU showed pronounced antitumor effects (Figure 7G,H). CD31 and PAS staining of the tumor tissue also showed that GCG@LFA+5‐FU significantly reduced blood vessels and vascular mimicry (Figure S10A–C, Supporting Information). Meanwhile, we examined the liver function by measuring the levels of serum transferases, which remained similar among all groups (Figure S10D, Supporting Information). In addition, hematoxylin and eosin staining showed no obvious changes in the lungs, liver, or pancreas (Figure S10E, Supporting Information). These data suggest the potential of a lipid‐based nanoparticle delivery system of glucagon as an adjuvant therapy drug for treating patients with CRC.

## 3 Discussion

A combination of multiple chemotherapeutics and antiangiogenic drugs is the first‐line treatment for advanced CRC.^[^ ^3^ ^]^ Standard treatment using folinic acid, 5‐FU, oxaliplatin, and irinotecan (FOLFOXIRI) plus bevacizumab significantly improves overall survival, but the response rate is only ≈40%.^[^ ^12^, ^39^ ^]^ To increase the drug response rate and efficacy, the combination of chemotherapy with other targeted therapies has been widely studied.^[^ ^40^, ^41^ ^]^ Efforts have been made to identify biomarkers to predict drug efficacy, such as growth factor expression, circulating cells, and tumor microenvironment.^[^ ^42^ ^]^ However, these candidates have not been successfully evaluated clinically.

Glucagon is a metabolism‐related hormone which is mostly studied in metabolic diseases such as diabetes, but not in relation to tumors. Glucagon may promote tumor angiogenesis by regulating the HIF‐VEGF axis.^[^ ^24^ ^]^ In addition, glucagon may potentially promotes tumor cell growth.^[^ ^23^ ^]^ However, its involvement and underlying mechanisms are still unclear. Therefore, it is important to clarify the role of glucagon in cancer development.

We carefully examined the effect of glucagon on tumor proliferation using both in vitro and in vivo models. Unexpectedly, glucagon did not promote colorectal cell proliferation, although western blotting revealed glucagon receptor expression in tumor cells. Interestingly, the glucagon receptor was expressed on endothelial cells, and the treatment of endothelial cells with glucagon resulted in the dose‐dependent inhibition of cell viability, migration, and tube formation. In contrast, VEGF rescued the glucagon‐induced inhibition of endothelial cells, suggesting that the role of glucagon in angiogenesis is VEGF‐dependent. There may be a crosstalk between glucagon and VEGF signaling. Downstream of the GCGR signaling molecule, cAMP can inhibit ERK signal transduction in connection with Ca^2+^ in renal cells, which is also the key cascade process during VEGF signal transduction.^[^ ^43^ ^]^ Thus, glucagon may function together with Ca^2+^ flux to inhibit ERK activity. However, further evidence and validation are required. Nevertheless, these results indicate that glucagon may inhibit tumor angiogenesis rather than promote vessel growth. In contrast, glucagon has a pro‐angiogenic function, indicating that glucagon plays different roles in different contexts. As reported earlier, the administration of high glucagon doses constantly elevates glucose levels favoring the Warburg effect in the tumor microenvironment, leading to the rapid proliferation of tumor tissue, including endothelial cells, but may not affect VEGF signaling, as direct evidence was missing in that report.^[^ ^23^ ^]^

Our exciting finding indicates the potential of glucagon as a novel endogenous antiangiogenic inhibitor which suppresses tumor growth. Glucagon only affected tumor vessels but did not significantly suppress tumor growth in our CRC mouse model, although an inhibitory trend was observed. This could be attributed to the short treatment period, dose effect, or other tumor environmental factors that circumvent angiogenesis and support tumor growth. Therefore, the glucagon‐induced antiangiogenic effect did not surpass the threshold of tumor inhibition. The underlying mechanisms require further investigation.

To our knowledge, this study is the first to report that glucagon affects vascular mimicry, along with exhibiting antiangiogenic potential. Surprisingly, glucagon reduced the number of pseudo‐vessels composed of tumor cells. Although the underlying mechanisms need further elucidation, one possibility is that glucagon impairs the migration of tumor cells, as shown in our in vitro model, thus making tube formation difficult for the tumor cells. Markers of vascular mimicry, such as MMP‐2, MMP‐9, and VE‐cadherin, decreased on glucagon stimulation, suggesting that glucagon suppresses vascular mimicry through these molecules in CRC cells.

Monotherapy with antiangiogenic drugs produces marginal clinical benefits in patients with cancer; however, combination with chemotherapy can produce a synergistic antitumor effect.^[^ ^22^, ^39^, ^44^ ^]^ Thus, glucagon could be considered as an antiangiogenic agent for combination therapy, which significantly inhibited tumor growth in our models. Data from HFD‐fed animals further confirmed the hypothesis that endogenous glucagon has the same effect on tumors. These results indicate that some diabetic patients with CRC might exhibit better therapeutic effects with 5‐FU because of increased glucagon levels.

Analysis of tumor vessels revealed that glucagon not only induced blood vessel regression, but also destructively impaired vessel structure and function. Electron microscopy and immunostaining revealed leaky and impaired endothelial cell membranes, increased permeability, and decreased perfusion. Therefore, tumor tissues with defective blood vessel networks are more vulnerable to chemotherapy. The destructive and permeable phenotype of vessels can also improve 5‐FU drug delivery into the tumor tissue, thus increasing the 5‐FU concentration and resulting in tumor cell inhibition. Glucagon can induce vessel permeability and destruction, thereby inducing further hypoxia and tissue starvation, leading to cell death. Usually, normalized vessels regain perfusion function, which can increase drug delivery efficiency, eventually improving the antitumor effect. However, in our settings, since 5‐FU itself also targets blood vessels, the more 5‐FU enters blood vessels, higher the possibility that blood vessels are destroyed by 5‐FU, suggesting that well‐perfused vessels are more vulnerable to the drugs. Therefore, it might be difficult to identify the small window in which we could observe significantly more well‐perfused vessels in the combination treatment group. Increased apoptosis was observed in the tumor tissues of the combination treatment group. Furthermore, macrophages and stromal cells significantly decreased after glucagon treatment (data not shown), indicating changes in the tumor microenvironment, which may also contribute to tumor inhibition.

Inhibition of glucagon signaling with the glucagon receptor inhibitor, AL, reversed the glucagon‐enhanced tumor suppression and blood vessel regression. This result demonstrated that the effect was mediated by glucagon receptors and downstream signaling. Further analysis of downstream signaling showed that glucagon inhibited the VEGF/VEGFR axis, leading to endothelial cell apoptosis and migration inhibition. However, the association between the glucagon receptor and the VEGF/VEGFR pathway needs to be further investigated using RNA‐seq, proteomics, or metabolomics approaches, which may help identify the key molecules involved. Although glucagon has demonstrated antiangiogenic potential, this requires further validation in different cancers or chemotherapeutic drug treatments. Moreover, its antiangiogenic potential requires careful evaluation for use in patients with cancer as systemic delivery of glucagon may disturb the balance of blood glucose levels and increase the risk of developing metabolic disorders. However, glucagon may also serve as a predictive marker of chemotherapy efficacy. Both, our findings and the TCGA data showed that high glucagon levels are associated with good overall survival among patients with CRC. Correlation analysis of clinical data revealed a positive link between glucagon expression and chemotherapy response in patients with CRC. Thus, monitoring glucagon levels may help select drug‐sensitive populations for chemotherapy. However, there are some limitations in this study which need further validation: 1. The in vivo model we used has a limited treatment window within the ethical framework. Longer treatment period with glucagon alone to the tumor model may exhibit a stronger antiangiogenic effect and finally resulting tumor growth retardation. 2. The detailed signaling map from GCGR to VEGFR is still not clear, and the molecular mechanisms are to be investigated. 3. Clinical trials are worth doing to translate this findings into clinical practice.

**Figure 8:**
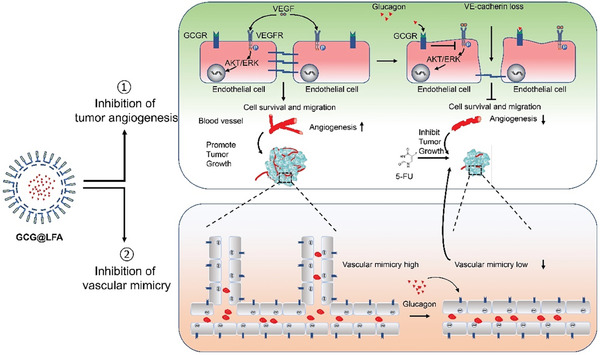
Schematic representation of the synergistic effects of glucagon and 5‐FU on tumor inhibition. Tumor‐derived vascular endothelial growth factor (VEGF) promotes angiogenesis via the VEGF/VEGFR‐AKT/ERK axis, which supports tumor growth and metastasis by stimulating endothelial cells. However, activation of the glucagon receptor on endothelial cells by lipid nanoparticle delivery (GCG@LFA) blocks VEGFR phosphorylation, which results in apoptosis, decreased migration of endothelial cells, and induces defects in VE‐cadherin loss and cell membrane destruction. Moreover, GCG@LFA also inhibited tumor vascular mimicry, further discrupting metabolic supply. Together with double inhibition, GCG@LFA impairs the tumor vessel network and facilitates the 5‐FU‐induced killing of vulnerable tumor cells.

In conclusion, glucagon could increase 5‐FU efficacy on tumor growth by inhibiting tumor angiogenesis, which blocked VEGFR phosphorylation and downstream signaling. Additionally, glucagon targeted vascular mimicry, which further blocked blood supply to the tumor tissue, leading to significant tumor suppression by dual disruption of the tumor vessel system (**Figure** **8**). Our data imply that glucagon may not only sensitize 5‐FU, but might also work with other chemotherapeutics, since glucagon‐induced vascular defects are a broad‐spectrum phenomenon. Moreover, patients with CRC and high glucagon levels may benefit from 5‐FU or other chemotherapeutic drugs. Patients with diabetes associated with CRC may have a better prognosis if high endogenous glucagon levels are present. A further interesting and potentially significant extension of this work is the liposome‐mediated tumor targeted delivery of glucagon and its acquired synergistic antitumor effect. However, further clinical investigations are warranted. In summary, our data provide evidence that glucagon contributes to tumor inhibition and may serve as a potential prognostic marker or therapy to enhance chemotherapy efficacy.

## 4 Experimental Section

### Reagents and Antibodies

The following antibodies were used for western blot analysis and immunochemical staining: anti‐CD31 Antibody (R&D, AF3628); anti‐Ki67polyclonal antibody (Proteintech, 27309‐1‐AP), anti‐CA9 polyclonal antibody (Proteintech, 11071‐1‐AP); anti‐cleavedcaspase‐3antibody (Servicebio, GB1153), anti‐beta actin (β‐actin)mouse monoclonal antibody (Servicebio, GB12001); anti‐VEGFR2 polyclonal antibody (Affinity, AF6281), anti‐phosphorylatedVEGFR1 (Tyr1213; p‐VEGFR1) antibody(Affinity, AF3204), phospho‐VEGFR2 (Tyr951; p‐VEGFR2) antibody (Affinity, AF3281), Akt antibody (Affinity, AF6261), phospho‐pan‐AKT1/2/3 (Thr308; p‐Akt) antibody (Affinity, AF3262), ERK1/2 (Erk) antibody (Affinity, AF0155), Phospho‐ERK1/2 (Thr202/Tyr204; p‐Erk) antibody (Affinity, AF1015); Anti‐VEGF Receptor 1 (VEGFR1) antibody (Abcam, ab32152); anti‐PDGFR‐β antibody (Abcam, ab32570); anti‐glucagon antibody (Abcam, ab92517); Anti‐Beta tubulin (β‐tubulin) antibody (Bioss, bs‐0715R); anti‐GAPDH antibody (CUSABIO, CSB‐PA00025A0Rb); anti‐PDGF‐BB antibody (Wanleibio, WL01625); anti‐tumor necrosis factor α (TNF‐α) antibody (Wanleibio, WL01581); anti‐FGF2 antibody (Wanleibio, WL03542); anti‐IL6 antibody (Wanleibio, WL02841); MMP9 polyclonal antibody (Proteintech, 27306‐1‐AP); MMP2 polyclonal antibody (Proteintech, 10373‐2‐AP); Alexa Fluor 647‐AffiniPure donkey anti‐mouse IgG (Jackson, 705‐605‐003); Alexa Fluor 488 Goat anti‐rabbit IgG (Abbkine, A22120). Glucagon (MB11250) was from Meilunbio. FITC‐dextran 2000(FD2000) was purchased from TdBLabs. FITC‐dextran 70 (D1822) was from ThermoFisher Scientific. Hydrogenated soy phosphatidylcholine (HSPC, 92128‐87‐5) and cholesterol (57‐88‐5)were purchased from AVT (Shanghai) Pharmaceutical Tech Co., Ltd. DSPE‐PEG2000‐FA (Ruixibio, R‐0043) was commercially available from Xi`an Ruixi Biological Technology Co., Ltd.. Fluorescein 5(6)‐isothiocyanate (FITC; 27072‐45‐3) was bought from Shanghai Aladdin Biochemical Technology Co., Ltd.. Cell membrane staining dye 1,1′‐dioctadecyl‐3,3,3′,3′‐tetramethylindocarbocyanine perchlorate (Dil; 41085‐99‐8) was bought from Beyotime Biotechnology. Recombinant VEGF165 Protein(abs00899) was purchased from absin. Anti‐VEGF neutralizing antibody (QL1101) was bought from Qilu pharmaceutical co.,ltd. Puromycin dihydrochloride hydrate (A610593) and DH5α competent cells(B528413) was bought from Sangon Biotech. Lipofectamine 3000 (L3000015) was purchased from Thermo Fisher Scientific. Plasmid Extraction Kit (DP104) was TIANGEN Biotech (Beijing). other agents included oxaliplatin (selleck, S1224) and Calcium Folinate (Meilunbio, MB1043).

### Clinical Sample Collection and Preparation

Six matched pairs of colorectal cancer and normal mucosal tissues were collected by the same surgeon at the Affiliated Yantai Hospital of Binzhou Medical University. The microarray data (product number BC05118c) was purchased from U.S. Biomax, Inc., containing 82 paired tumor and normal mucosal tissues (including parameters of Gender, Age, Grade, TNM stages).

### Ethical Statement

All subjects gave their consent for inclusion before they participated in the study. The study was conducted in accordance with the Declaration of Helsinki, and the protocol was approved (permission number: 2016–019) by the Human Research Ethics Committee of Binzhou Medical University (Yantai, China).

### Sample Preparation for Proteomics

The tumor and normal mucosal tissues were suspended and homogenized in lysis buffer (9 m Urea, 20 mm HEPES, and 1 mm cocktail). A sample corresponding to 20 µg digested proteins was desalted using C18 Stage‐tips with EmporeDisksC18 from Varian (Palo Alto, CA, USA), and dried completely in a vacuum centrifuge. The mass spectrometry proteomics data have been deposited to the Proteome Xchange Consortium via the PRIDE partner repository with the dataset identifier PXD009475.

### Immunohistochemistry (IHC) Staining and IHC Score Analysis

The slices were baked in a 60 °C oven for 2 h, and then in xylene and alcohol to remove paraffin. Antigen retrieval was performed by soaking the slices into the 0.01 m sodium citrate buffer in a microwave oven (for 5 min at high heat and 25 min at low heat), then naturally cool down to room temperature. Rinse with phosphate‐buffered saline (PBS; 20 mm Na‐phosphate, 150 mm NaCl, and 0.05% Tween 20, pH 7.4) for 3 times, 10 min each and incubate with 3% H_2_O_2_ for 10–15 min, with 0.2% TritonX‐100 for 10 min, with goat serum at 37 °C for 30 min, and with the first antibody overnight at 4 °C, gradually. After washing PBS three times, the slices were added the secondary antibodies and incubated at 37 °C for 30 min. The following step was the working DAB solution was added onto the samples for reaction. Soaking slices into the distilled water to terminate the DAB reaction. For PAS staining, 0.5% periodic acid was added and incubated for 5–8 min. Wash with double distilled water (ddH_2_O) for three times. Add Schiff staining buffer, incubated for 10–20 min. Rinse with ddH_2_O for 5 min, stain with hematoxylin solution dropwise for 5 min, and rinse with tap water for 10 min. Differentiate with acidic differentiation solution for 2–5 s and rinse with tap water for 10 min. Dehydrate the slide and air dry it. After dropping resin gel film, observe and collect pictures under inverted microscope. Two researchers were blindly assigned to review the slides and score the staining. Patterns of tissue section staining were scored according to the following standards: staining intensity was classified as 0 (lack of staining), 1(mild staining), 2 (moderate staining) or 3 (strong staining); the percentage of staining was designated 1 (< 25%), 2 (25–50%), 3 (51–75%), or 4 (> 75%). For each section, the score was calculated by multiplying these two values (which ranged from 0–12)

### Mouse Tumor Models

The BALB/c male mice were purchased from the SPF (Beijing) Biotechnology Co., Ltd. CT26 tumor cells were subcutaneously injected into the right flank with 1 × 10^6^ cells in 0.1 mL of PBS per mouse. Then, the mice were randomized separated into different treatment groups: the control, glucagon, 5‐FU, glucagon+5‐FU, adomeglivant (AL; MCE, HY‐19904), or AL+glucagon+5‐FU, FOLFOX, glucagon+FOLFOX. When the average volume of tumors reached ≈200 mm^3^, treatment was started with intraperitoneal injection for PBS, 5‐FU (25 mg kg^−1^, every other day),injected with FOLFOX (5‐FU, 12.5 mg kg^−1^, i.p., every other day; Oxaliplatin 1.5 mg kg^−1^, i.v., every 4 days and Folinate 4.5 mg kg^−1^, i.v., every 4 days), glucagon (20 µg per mouse, daily), or gavage for AL (1 mg kg^−1^, daily). Each group was administered for about 14 days.

### Vessel Perfusion and Permeability Assay

Dextran 2000‐kDa used for perfusion or 70‐kDa for permeability assay were intravenously injected into tumor bearing mice and let the dextran circulating for 15 min or 5 min respectively. Then sacrifice the mice and tumor tissues and blood samples were collected for later analysis. Tumor tissues were dissected out and fixed in 4% paraformaldehyde at 4°C overnight. Tumor tissue between the peripheral and central necrotic area were stained with CD31 by whole mount staining method. Images were taken using a confocal microscope (Zeiss LSM880with Airyscan, Germany). All procedures have been approved (approval no. 2021–201) by the Animal Ethics Committee of Binzhou Medical University.

### Patient‐Derived Xenograft

The tumor models generated by implantation of fresh human colorectal tumor tissue into immunodeficient mice (NSG, purchased from Beijing Vitalstar Biotechnology Co.,Ltd.). When tumor volumes reached ≈200 mm^3^, pdx mice were randomized into four groups for the treatment of Vehicle, glucagon, 5‐FU, or 5‐FU+glucagon respectively.

### High‐Fat Diet (HFD) Mouse Models

The HFD groups were fed a High‐fat diet(60%fat), whereas the Normal‐chow diet (NCD)group was fed a basal diet (16%fat) for 12 weeks. Then mice were also divided into four groups: NCD, HFD, NCD‐5FU and HFD‐5FU with CT26 tumor inoculation. When the average volume of tumors reached ≈200 mm^3^, treatments were started with intraperitoneal injection for PBS and 5‐FU.

### Transmission Electron Microscopy (TEM)

Fresh tumors were fixedin2.5% glutaraldehyde, then dissected into less than 1 mm^3^. Samples were rinsed with 0.1 m phosphoric acid rinse solution and fixed with 1% osmic acid fix solution. Samples were then dehydrated with a stepwise ethanol gradient, placed in acetone, embedded in Epon 812 and processed for sectioning on an ultramicrotome (LEICA, EMUC7). Ultrathin sections (≈70 nm) were stained with uranyl acetate and lead citrate for 15 min and observed on a transmission electron microscope (JEOL, JEM‐1400) at 100 KeV.

### Whole‐Mount Staining

Fresh tumors were fixed in 4% PFA. Then they were cut into thin pieces, digested with protease K(10 mm), permeabilized with 100% methanol, blocked and incubated with antibodies. The stained tissues were mounted with Vectashield mounting medium (H‐1000, Vector Laboratories, USA). Fluorescent signals were examined with a confocal microscope (Zeiss LSM880with Airyscan, Germany).

### Isolation of Endothelial Cell from Tumor Tissue

Endothelial cells were isolated from CT26 tumor tissue by magnetic activated cell sorting. In brief, CT26 tumor bearing mice was sacrificed by cervical dislocation when tumor sized reached 0.6–1.0 cm^3^. Tumor tissues were dissected and cut into small pieces. Single cell separation was processed by collagenase digestion followed with trypsin digestion. First, oscillate the tumor pieces at 1700 rpm in 0.15% collagenase (C2674, Sigma‐Aldrich) followed by incubation at 37 °C for 5 min. This oscillation‐incubation cycle was repeated 10 times. Tissue pieces were then centrifuged at 300 g for 6 min and discarded the supernatant. Wash with 20 mL PBS twice. Digest the tissues again with 10 mL 1% trypsin and incubate for 10 min. Stop the digestion by adding 2 mL fetal bovine serum (FBS). Filter the tissue‐cell mixture using 40 µm cell strainer (352 540, FALCON), and centrifuge briefly, wash with PBS again and collect the single cell pellet. Add 5 mL red blood cell Lysis Buffer into the cell precipitation and incubate the samples at room temperature for 5 min. Stop the reaction with 5 times volume of PBS and centrifuge to obtain the cell pellet. Resuspend the cells with 1 mL 0.5% bovine serum albumin (BSA) (00‐4333, Invitrogen), mix well for 15 min. To enrich the CD31^+^ endothelial cells, CD45 magnetic microbeads (130‐052‐301, Miltenyi Biotec) were applied first to exclude the CD31^+^ leukocyte subtypes and platelets, and then CD31 microbeads (130‐097‐418, Miltenyi Biotec) were followed to enrich the CD31^+^ endothelial cells according to the manufacturer's instructions. Briefly, cells were incubated with 10 μl CD45 antibody labeled magnetic microbeads and 90 μL 0.5% BSA buffer for 1 × 10^7^ cells, mix for 15 min. Apply the cell suspension onto the prepared MS columns (130‐042‐201, Miltenyi Biotec), collect flow‐through containing unlabeled cells. Next, repeat the incubated steps with CD31 microbeads, and wash the column with degassed buffer. Add 1 mL buffer to flush out the CD31^+^ endothelial cells by applying the plunger. The cells are ready to be used for later experiments.

### Isolation of Pancreatic Islets from Mouse Pancreas

Mice were sacrificed and pancreas were dissected out. Remove adipose tissue and inject 0.5 mg mL^−1^ collagenase P (11 249 002 001,Roche Diagnostics) to fully inflate the pancreas. Digest the pancreas at 38 °C for 10 min in a Eppendorf tube (shake the tube every 1 min after 5 min pre digestion). Shake the pancreas until the appearance to be sandy like appearance. Transfer the pancreas into cell culture dish with pre‐chilled Hanks buffer (MA0039, Meilunbio) to stop digestion reaction. Collect the pancreatic islets under stereomicroscope for following experiments.

### Extraction of Total RNA

4T1 cells or isolated endothelial cells were collected by centrifuge, and the cell pellet was mixed with 1 mL of Trizol, and let stand at room temperature for 5 min. Then, 200 µL chloroform was added. After vortex oscillation, let it stand at room temperature for 5 min. After centrifuge (4 °C, 12 000 rpm) for 15 min, transfer the supernatant to a new Eppendorf tube, followed by mixing with an equal volume of isopropanol. Then, the mixture was centrifuged and the sedimentation was retained and repeatedly washed with pre‐cooled 75% anhydrous ethanol by centrifugation (4 °C, 7500 rpm) for three times. Lastly, discard the supernatant and dry the RNA precipitate at room temperature. Diethyl pyrocarbonate (DEPC)‐treated water was used to dissolve RNA. RNA concentration was determined by a Nano Ready spectrophotometer (Life Real, FC‐1100).

### Quantitative PCR

Reverse transcription of total RNA to cDNA was performed using the reverse transcriptase (R323‐010, Vazyme). RT‐qPCR was performed using SYBR qPCR Mix (Q711‐02, Vazyme) with eppendorf . Primer sequences used were as follows:

mouse gcgr‐F: 5′‐TTGGTACCACAAAGTGCAGC‐3′;

mouse gcgr‐R: 5′‐TGCAGCAGCTCCCACTC‐3′;

actin‐F:5′‐GCCGACAGGATGCAGAAGGAGATCA‐3′;

actin‐R:5′‐AAGCATTTGCGGTGGACGATGGA‐3′.

Relative quantitation of gene expression was calculated using the 2−ΔΔCT method.

### Measurement of Glucose

Blood samples were collected from tumor bearing mice by tail vein sampling and glucose were measured using glucose reader (Andon, AG‐605). Briefly, the tip of mouse tail was cut off and discard the first drop of the blood, then take the send drop of blood to the testing paper, and read the glucose value.

### Western Blotting

Total 40 µg protein was separated and transferred to polyvinylidene fluoride (PVDF) membrane (Millipore), which was then incubated with appropriate antibodies. Protein expression was detected using Affinity ECL Kits (KF001, Affinity Biosciences) and photographed in a chemiluminescence instrument (Clinx science, 3100Mini, 90 175).

### Cell Culture

CT26 and 4T1 were purchased from ATCC company and cultured with RPMI 1640 medium containing 10% FBS. Human colorectal cancer cell line SW480 were purchased from School of Basic Medicine Peking Union Medical College and cultured with DMEM containing 10% FBS. Mouse vascular endothelial C166 cells were purchased from Shanghai Zishi Biotech Co., Ltd. and cultured with DMEM containing 10% FBS. Cell proliferation assay was measured by using CCK8 kits. The absorbance of the solution was measured on a Bio‐Rad 680 microplate reader at 450 nm.

### GCGR‐Knock Down and GCG‐Overexpressing Cell Lines Establishment

To knock down GCGR or over‐express GCG (OE‐GCG), the shRNA targeting GCGR (5′CCACAGTGATCATGCAGTATTCAAGAGATACTGCATGATCACTGTGG 3′) was constructed into pLKO.1 vector and the OE‐GCG was constructed into pLVX vector by using the empty vector as control. These pLKO.1/ pLVX constructs and control vector were then individually transfected into HEK293T cells together with Δ8.9 and VSVG for lentivirus generation using Lipofectamine 3000 (vector: Δ8.9:vsvg = 12:6:1.5). At 48 h after transfection, the culture medium containing lentivirus particles was collected and treated by centrifugation at 12 000 g for 5 min to get rid of the cell debris before use. For the establishment of CT26 cell model, The cells (≈70% confluence) were incubated with culture media containing lentiviral particles and 4 µg mL^−1^ polybrene at 37 °C incubator with 5% CO_2_ for 48 h, and 7.5 µg mL^−1^ puromycin were used to select the infected cells.

### Cell Migration and Tube Formation Assay

The trans‐well migration assay system (8 µm;Corning, 3422) was used for cell migration assay. C166 and CT26 Cells are placed inside the upper chamber. Migratory cells are stained and counted. Cultrex Basement Membrane Extract (BME, Cat. No.3432, Roche) was used for tube formation assay. The formation of tube‐like structures was observed and photographed under microscope (U‐LS30‐3, Olympus).

### Flow Cytometry Analysis

The cell apoptosis analysis was measured by using the Annexin V‐FITC/PI double staining kit (Cat. NO: KGA108, KeyGEN BioTECH, China). The fluorescence was detected by flow cytometry.

### Enzyme‐Linked Immunosorbent Assay (ELISA)

CRC patient tumor tissue and paracancerous tissue samples were collected and homogenized by lysis buffer (RIPA buffer with 1% protease/phosphatase inhibitors) for ELISA assay. Human glucagon was detected using commercially available ELISA kits (ML bio, ml022730). All procedures were performed according to the manufacturer's instructions.

### Preparation of GCG@LFA and FITC/GCG@LFA

The components of liposomes were consisted of HSPC (92128‐87‐5, AVT, China), cholesterol (57‐88‐5, AVT, China) and DSPE‐PEG2000‐FA(R‐0043, Ruixibio, China) (85:10:5, molar ratio(%)). All liposomes were prepared according to the reported method with some modifications.^[^ ^28^ ^]^ Briefly, the lipids were dissolved in chloroform (67‐66‐3, YUANDONG, China), the solvent was evaporated under vacuum by a rotator Hei‐VAP Core (HeiDolph, Hei‐VAP, Germany) and a thin film was formed. Liposomal vesicles, PEGylated liposomes with tumor targeting folic acid molecules (LFA), GCG@LFA, FITC and GCG co‐loading PEGylated liposomes with tumor targeting folic acid molecules (FITC/GCG@LFA) were obtained by ultrasound with aqueous solution, GCG solution or FITC + GCG solution for 10 min at room temperature. The encapsulated molecules were removed by dialysis. Thus, tumor targeting liposomal nano system GCG@LFA and FITC/GCG@LFA was acquired. The encapsulation efficiency (EE %) of liposomes was expressed according to the following equation:

(1) $$\mathrm{EE}\%=\frac{W_{\mathrm{total}}-W_{\mathrm{free}}}{W_{\mathrm{total}}}\times 100\%$$

where W_free_ was the amount of not entrapped GCG and W_total_ represented the total amount of GCG. The particle size distributions were measured by Nano‐particle Zeta potential and absolute molecular weight analyzer (Malvern, U.K.). Liposome suspension was dropped onto a carbon film copper grid, followed by negative staining procedure using phosphotungstic acid and finally observed under a transmission electron microscope (JEOL, JEM‐1400, Japan). A LAMBDA 365 UV/Vis spectrometer (Perkinelmer, U.K.) was employed to obtain UV/Vis spectra of GCG, LFA, or GCG@LFA. Fourier transform infrared (FT‐IR) spectra were obtained on a Nicolet iS20 fourier transform infra‐red (FTIR) spectra (Thermo Scientific, U.K.) from 4000 to 400 cm^−1^ .

### Tumor Cell Targeting Assay

FITC/GCG@LFA particles were employed to evaluate the binding affinity of FA‐modified liposomes with tumor cells expressing folic acid receptors. Briefly, CT26 cells were plated at a concentration of 3×10^5^ cells in the confocal dish 24 h prior to the assay. Then, the cells were treated with folic acid (1 mm), FITC/GCG@LFA (equal valence to GCG (100 nm) and folic acid plus FITC/GCG@LFA for 6 h, respectively. After that, the cells were stained with cell membrane staining reagent Dil (10 µm) for 20 min and washed with PBS buffer for 3 times, followed by the observation under a laser scanning confocal microscope (Zeiss, Germany).

### H&E Staining

The sections were dewaxed in Xylene for 2–3 min, rehydrated in alcohol (100%, 95%, 70%) and brought into distilled water. Next, the nuclei were stained with hematoxylin for 5 min, sections were transferred into the differentiation fluid (1% hydrochloric acid alcohol) for 30 seconds, and stained with eosin for 30 second, and then were rinsed in running tap water for 10 min, respectively. Dehydration in alcohol (70%, 95%, 100%) and transparency in xylene were following rinsing. After drying in fume hood, sections were sealed with resinene.

### Statistics Analysis

The protein expression profiling, IHC staining, tube formation and transwell results were analyzed with the ImageJ software. Vessel and FITC perfused area were measured using the photoshop software. Statistic analysis were performed using the two‐tailed paired/unpaired Student's *t*‐test in GraphPad Prism 10.0.2 software (GraphPad Prism Inc., San Diego, CA, USA; http://www.graphpad.com), respectively. The logistic regression analysis was done with 18 CRC patients’ data (at late stage (T3+T4)) from the GEO dataset GSE69657 with the GraphPad Prism 10.0.2 software. P < 0.05 was considered to be significant.*, *P* < 0.05; **, *P* < 0.01; ***, *P* < 0.001. Error bars represent the standard error of the mean (SEM).

## Conflict of Interest

The authors declare no conflict of interest.

## Author Contributions

Y.X. and F.N. contributed equally to this work. Y.Z., G.T., P.W. generated the ideas, designed the experiment, supervised the project. Y.X. and F.N. performed most of the experiment. D.S., Y.P., Y.Z., X.W., S.L., X.Q., M.L., Y.Z., M.Y., C.Y., Y.Y., and B.A., helped with sample, data collection. X.K. and C.Z. analyzed clinical data. C.Y. provided valuable reagents, equipment, assisted with supervision. G.Z. and W.J. provided valuable discussion. J.M. analyzed proteomics data. C.Y., S.Z., and X.C. provided clinical samples. Y.Z., Y.X., P.W., and F.N. analyzed the data. Y.Z. and Y.X organized the figures, wrote the manuscript.

## Supporting information

Supporting Information

## Data Availability

The data that support the findings of this study are available from the corresponding author upon reasonable request.
