# Supplementary material for: Glucagon Enhances Chemotherapy Efficacy By Inhibition of Tumor Vessels in Colorectal Cancer
Source: Adv Sci (Weinh). 2023 Dec 10;11(6):2307271. doi: 10.1002/advs.202307271 (PMC10853751; doi:10.1002/advs.202307271)
Supplement: Supplementary file 1 — Supporting Information [file ADVS-11-2307271-s001.pdf]

ADVANCED SCIENCE
Open Access

Supporting Information

for *Adv. Sci.*, DOI 10.1002/advs.202307271

Glucagon Enhances Chemotherapy Efficacy By Inhibition of Tumor Vessels in Colorectal Cancer

Yuxue Xu, Feixue Ni, Daxi Sun, Yue Peng, Yaxuan Zhao, Xiaojun Wu, Shasha Li, Xiangyu Qi, Xinkang He, Min Li, Yizi Zhou, Chao Zhang, Miao Yan, Cuifang Yao, Shuaishuai Zhu, Yang Yang, Baijiao An, Chunhua Yang, Guilong Zhang, Wenguo Jiang, Jia Mi, Xinju Chen, Pengfei Wei, Geng Tian* and Yin Zhang**

Supporting Information

Glucagon enhances chemotherapy efficacy by inhibition of tumor vessels in colorectal cancer

Yuxue Xu[1,2#], Feixue Ni[1,2#], Daxi Sun[1,2], Yue Peng[1,2], Yaxuan Zhao[1,2], Xiaojun Wu[1,2], Shasha Li[1,2], Xiangyu Qi[1,2], Xinkang He[3], Min Li[1], Yizi Zhou[1], Chao Zhang[1], Miao Yan[1,2], Cuifang Yao[1,2], Shuaishuai Zhu[4], Yang Yang[1,2], Baijiao An[1,2], Chunhua Yang[1,2], Guilong Zhang[1,2], Wenguo Jiang[1,2], Jia Mi[1,2], Xinju Chen[4], Pengfei Wei[*1,2], Geng Tian[*1,2], Yin Zhang[*1,2]

Table S1. Glucagon expression in tumor and colon tissues

	Number	Glucagon expression		Chi-square (χ^2) value	p-value
		Low (%)	High (%)		
Tumor tissue	82	31.7	68.3	15.530	0.000081
Colon tissue	82	7.3	92.7		

Table S2. Correlation between glucagon levels and the clinical characteristics of patients with colorectal cancer

Variables	Patients	Glucagon expression		Chi-square	p-value
	(n =90)	Low (n=29)	High (n=61)	(χ^2)	
**Sex**					
Male	46	19	27	0.776	0.378
Female	44	10	34		
**Age** (years)					
≤ 70	53	22	31	5.091	0.024
> 70	37	7	30		
**TNM stage**					
Stage I/II	59	14	45	5.658	0.017
Stage III	31	15	16		
**Grade**					
1–2	68	18	50	4.214	0.040
3–4	22	11	11		

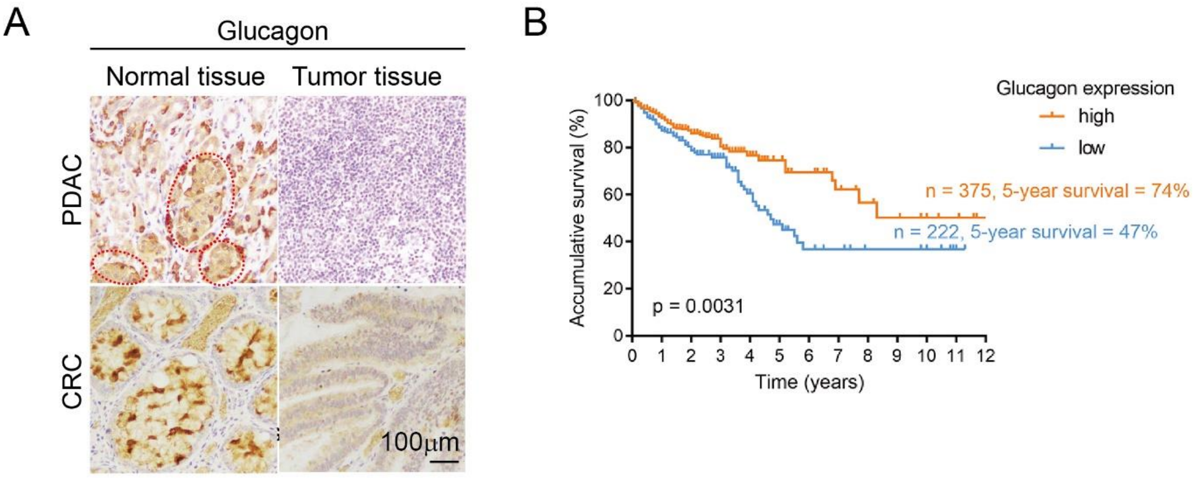

Figure S1

Figure S1. Glucagon expression in normal and tumor tissues. (A) Immunohistochemical staining for glucagon in patients with pancreatic and colorectal cancers. The red dotted circles represent the pancreatic islets (n=3). (B) Survival curve of patients with CRC with high glucagon versus low glucagon expression levels from the TCGA database.

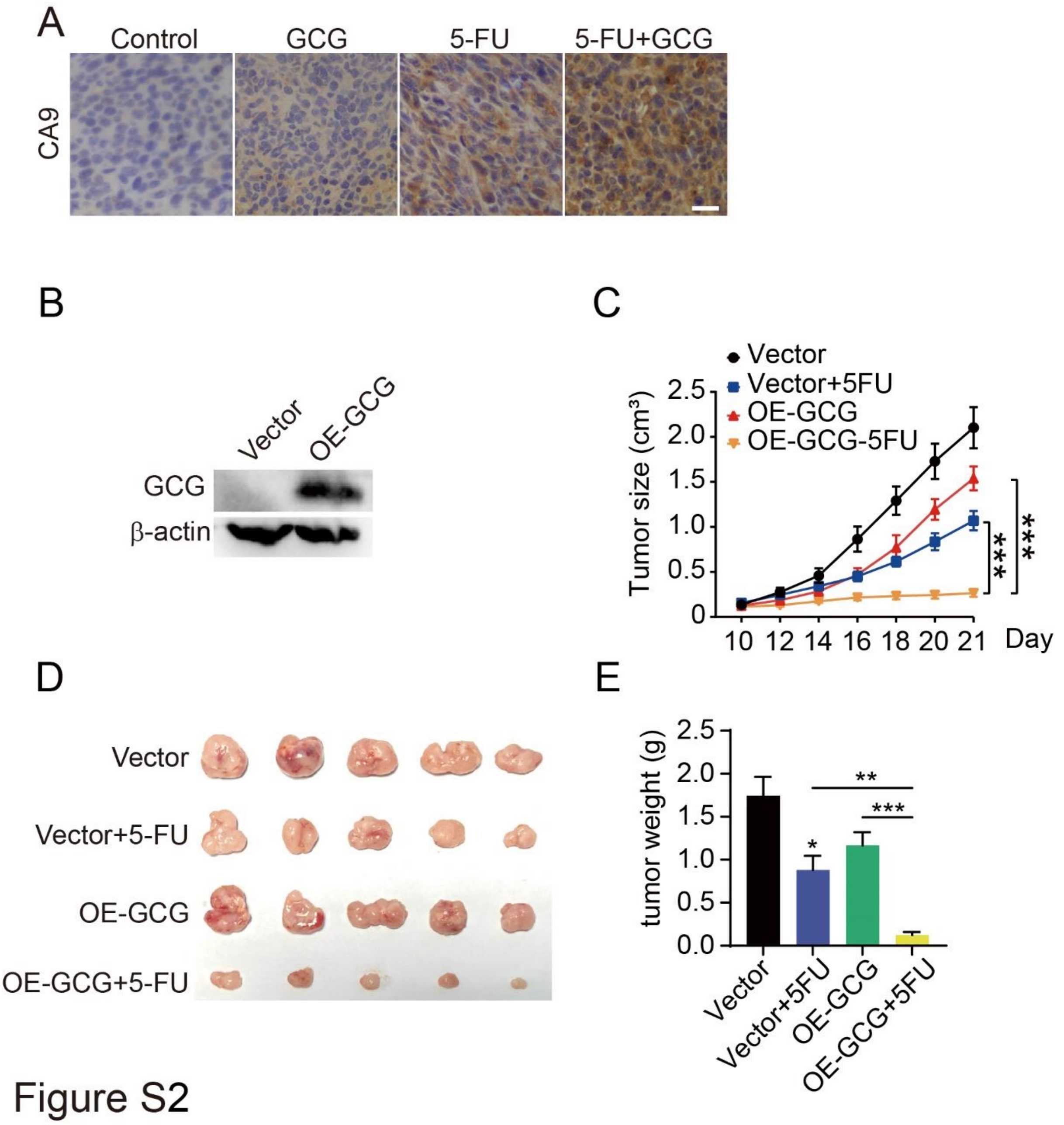

Figure S2. Glucagon induced tumor hypoxia and glucagon overexpression tumor model. **(A)** IHC staining of hypoxia marker CA9. Bar=100 μm. (n=5) **(B)** Glucagon expression in vector-transfected CT26 cells and glucagon-overexpressed CT26 cells **(C)** Tumor growth of CT26 and glucagon overexpression in CT26 cells. 5-FU, 25 mg/kg (n=5) **(D)** Images of CT26 tumors (n=5) **(E)** Quantification of CT26 tumors (n=5)

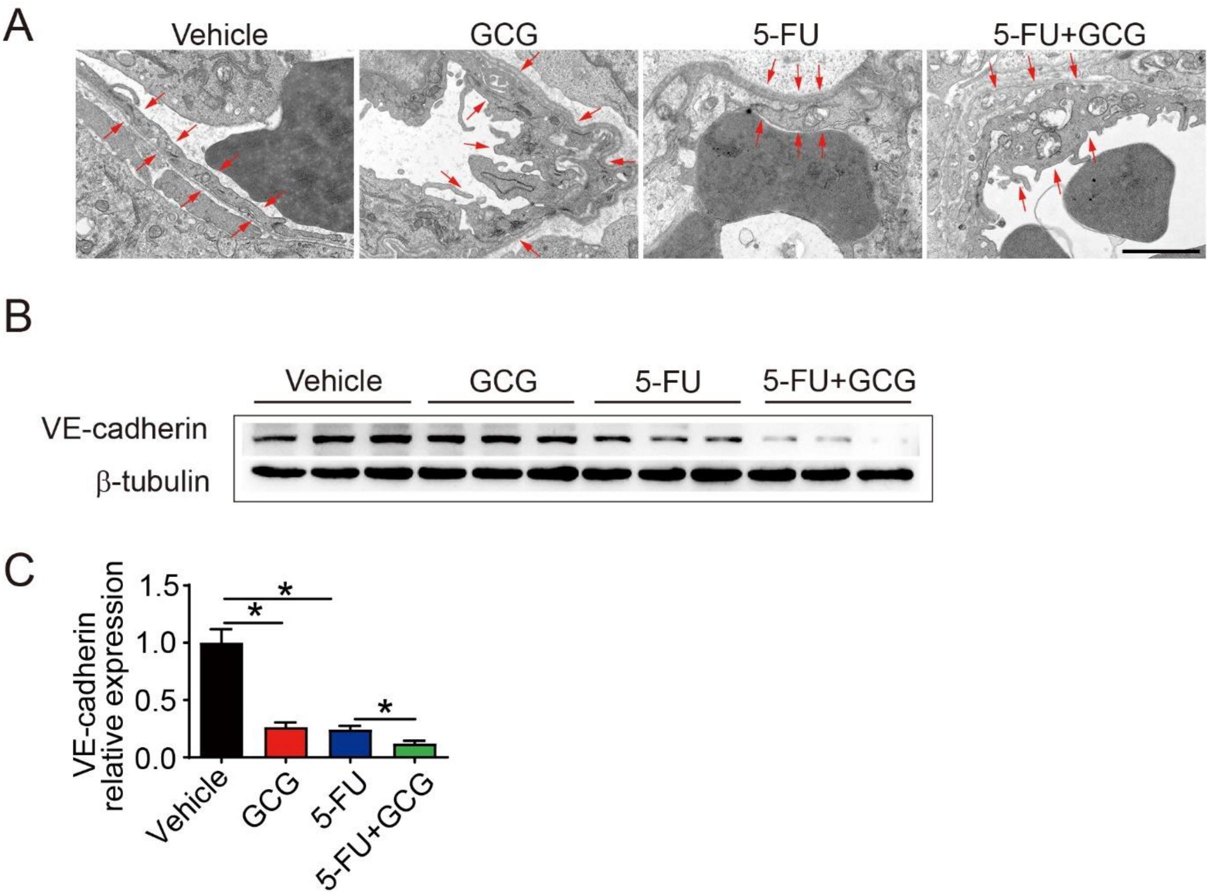

Figure S3

Figure S3. Glucagon induced endothelial cell structure changes (A) Electron microscopy images of endothelial cell membrane microstructure. Red arrows indicate the endothelial cell membranes. Bar: 2μm (n=14–16) **(B)** VE-cadherin expression in tumor tissues under different treatment conditions (n=3) repeated twice. **(C)** Quantification of VE-cadherin expression (n=3)

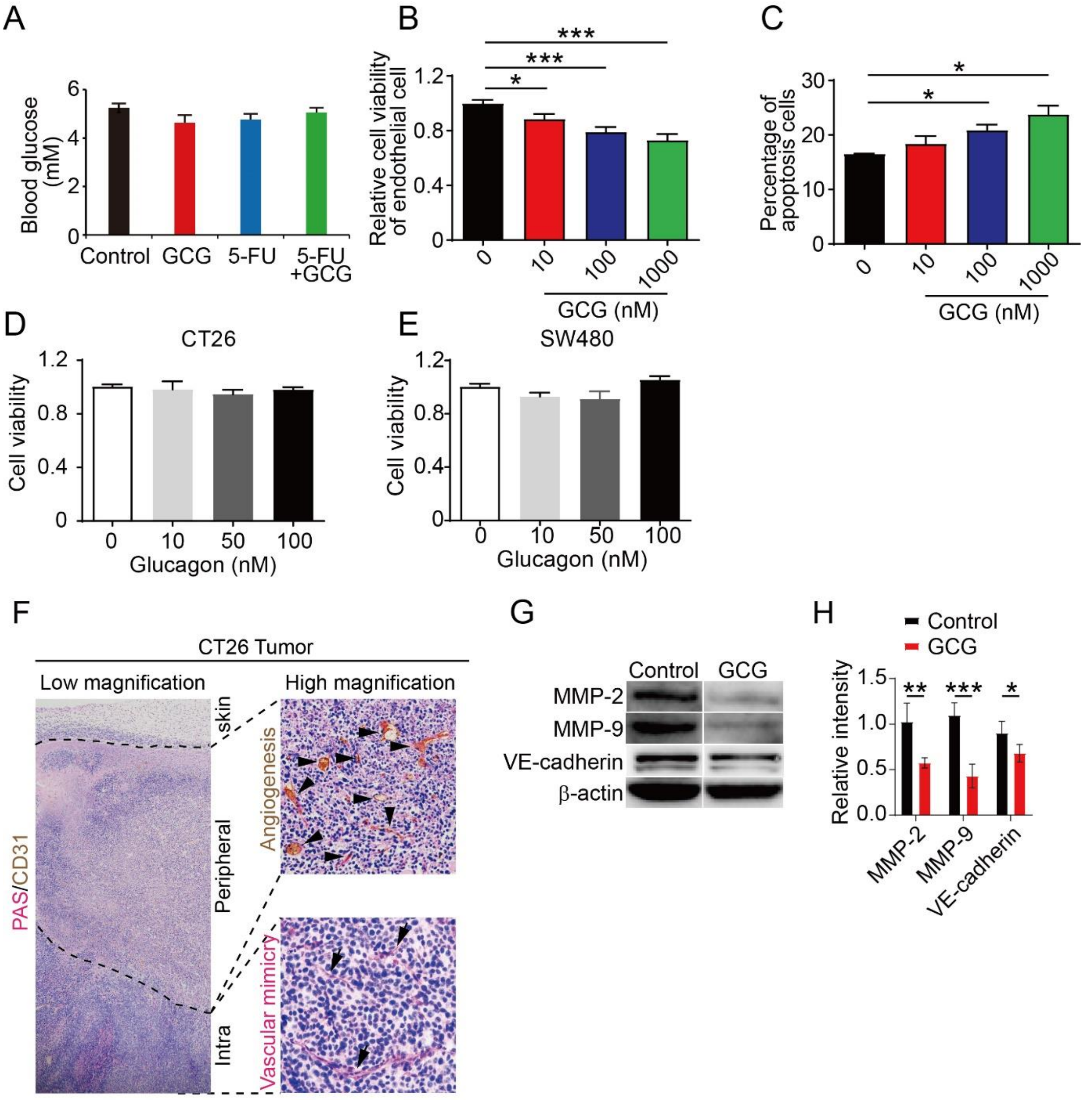

Figure S4

Figure S4. Glucagon directly targets endothelial cells and vascular mimicry. (A) Blood glucose levels in tumor bearing mice (n=5–7) **(B)** Quantification of endothelial cell viability (n=3) **(C)** Quantification of endothelial cell apoptosis (n=3) **(D)** Cell viability of CT26 treated with glucagon (n=3) **(E)** Cell viability of SW480 cells treated with glucagon (n=3) **(F)** PAS/CD31 staining of CT26 tumor **(G)** VM-related protein expression in CT26 tumor cells treated with glucagon (10 nM); the experiment was repeated three times. **(H)** Quantification of the relative expression levels of MMP-2, MMP-9, and VE-cadherin (n=3).

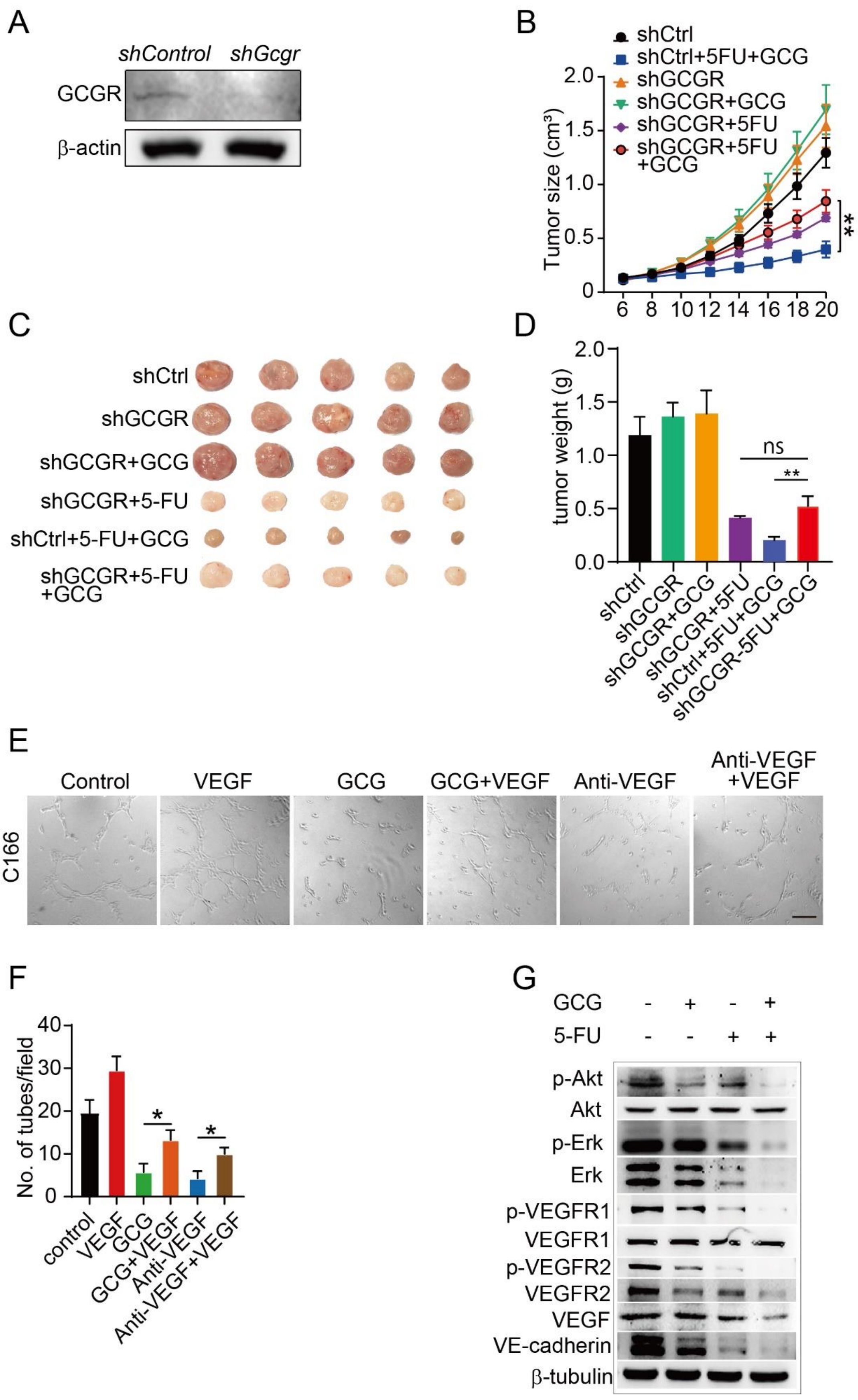
A
shControl shGcgr
GCGR
β-actin
B
shCtrl
shCtrl+5FU+GCG
shGCGR
shGCGR+GCG
shGCGR+5FU
shGCGR+5FU +GCG
Tumor size (cm³)
**
C
shCtrl
shGCGR
shGCGR+GCG
shGCGR+5-FU
shCtrl+5-FU+GCG
shGCGR+5-FU +GCG
D
tumor weight (g)
ns
**
shCtrl
shGCGR
shGCGR+GCG
shGCGR+5FU
shCtrl+5FU+GCG
shGCGR-5FU+GCG
E
Control
VEGF
GCG
GCG+VEGF
Anti-VEGF
Anti-VEGF +VEGF
C166
F
No. of tubes/field
control
VEGF
GCG
GCG+VEGF
Anti-VEGF
Anti-VEGF+VEGF
G
GCG
5-FU
p-Akt
Akt
p-Erk
Erk
p-VEGFR1
VEGFR1
p-VEGFR2
VEGFR2
VEGF
VE-cadherin
β-tubulin

Figure S5

Figure S5. The antiangiogenic effect of glucagon is dependent on glucagon receptor and VEGF signaling (A) Glucagon receptor expression in control and glucagon receptor knockdown CT26 cells. **(B)** Tumor growth in glucagon receptor-knockdown CT26 cells under different treatment conditions. GCG, 20 μg/mouse; 5-FU, 25 mg/kg (n=5). **(C)** CT26 tumor images. **(D)** Quantification of tumor weight (n=5) **(E)** Tube formation assay of endothelial cells challenged with VEGF and glucagon. VEGF, 8 ng/mL; GCG, 10 nM; bevacizumab, 250 μg/mL. The experiment was repeated three times. **(F)** Quantification of number of tubes in E (n=3) **(G)** Western blotting of the VEGF/VEGFR signaling pathway. GCG, 10 nM; 5-FU, 100 ng/mL; this experiment was repeated three times.

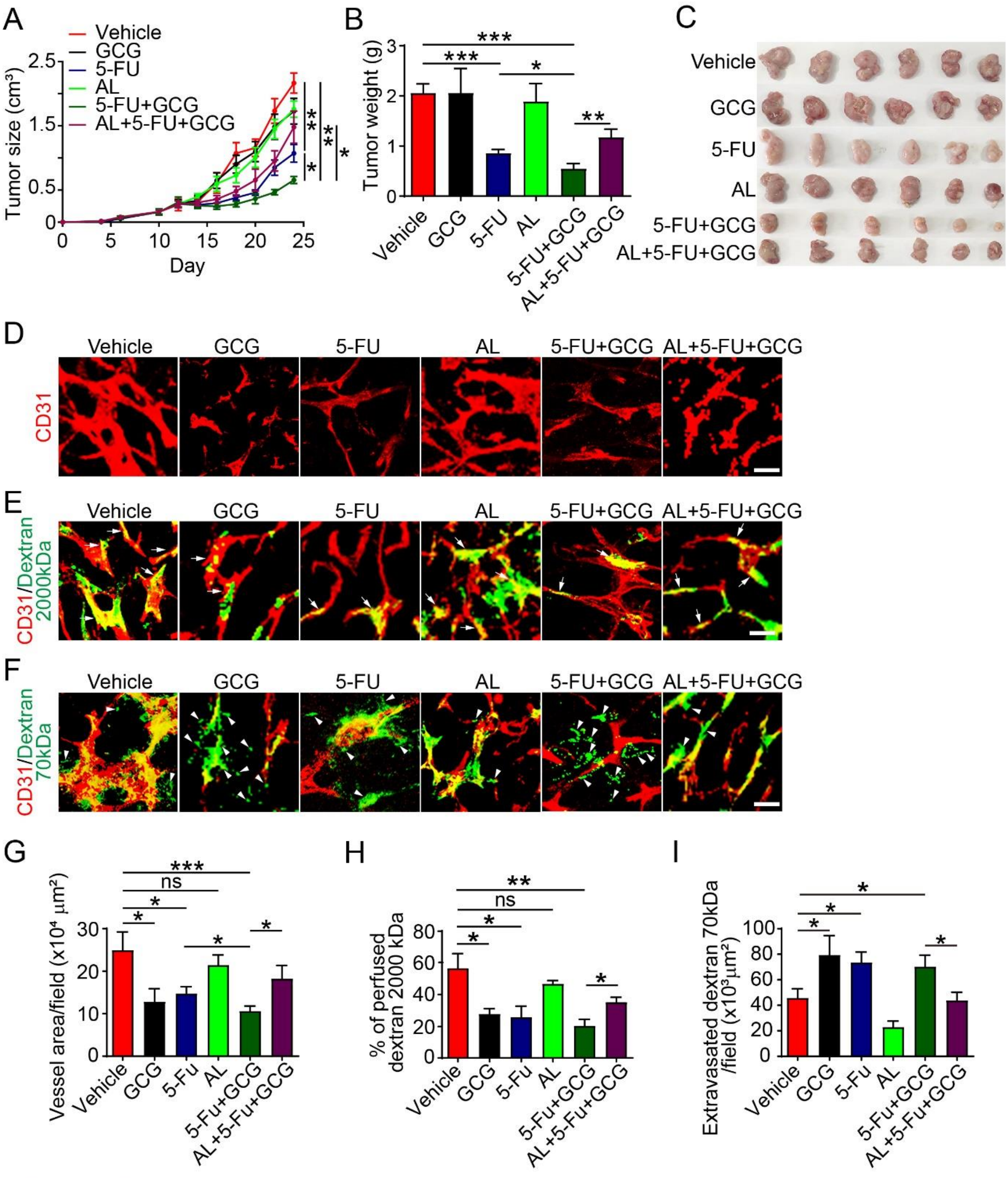

Figure S6

Figure S6. Glucagon-enhanced antitumor effect was reversed by inhibiting the glucagon receptor (A) Growth curves of CT26 tumors under different treatment conditions. Vehicle group, n=7; GCG (20 μg) and 5-FU (25 mg/kg) groups, n=8; AL (1 mg/kg) and 5-FU+GCG group, n=6; AL+5-FU+GCG group, n=9). The experiment was repeated twice. AL: adomeglivant. **(B)** CT26 tumor weight of different treatment groups. **(C)** CT26 tumor images from each treatment group **(D)** Blood vessel staining from different drug-treated CT26 tumor tissues. Bar: 20 μm **(E)** Perfusion analysis of tumor vessels in CT26 tumor tissues. Blood vessels are red, and dextran 2,000 kDa is green. The arrows indicate perfused vessels. Bar: 20 μm **(F)** Permeability analysis of CT26 tumor vessels using dextran 70 kDa. The blood

vessels are red, and dextran 70 kDa is green. Arrowheads indicate dextran leakage. Bar: 20 μm **(G)** Quantification of tumor vessels (n=6–9) **(H)** Quantification of perfused tumor vessels (n=6–9) **(I)** Quantification of extravasated dextran (n=6–9)

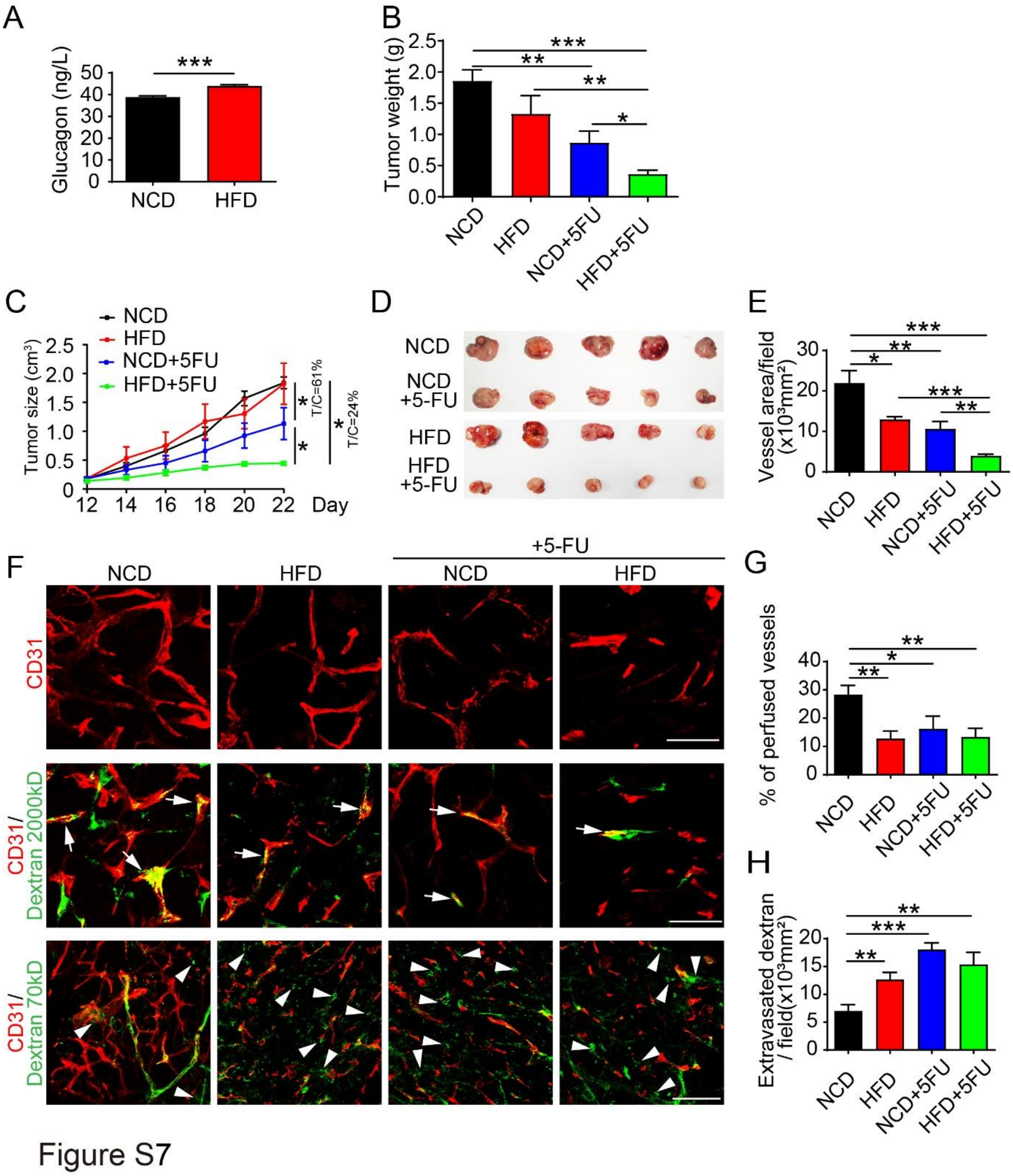

Figure S7. Endogenous glucagon may enhance 5-FU treatment in a diabetic mouse tumor model. **(A)** Serum glucagon levels (n=5–8) **(B)** Quantification of tumor weight in different groups. **(C)** Tumor growth of CT26 tumors on normal chow diet (NCD), high-fat diet (HFD), or 5-FU treatment; T/C: treatment/control. 5-FU, 25 mg/kg. (NCD and NCD+5-FU group, n=8; HFD and HFD+5-FU group n=6). **(D)** Tumor images from each group **(E)** Quantification of CD31 positive area in tumors (n=5) **(F)** CD31, dextran 2,000 kDa, and dextran 70 kDa staining of tumor tissues. Bar 100 μm (n=5) **(G)** Quantification of perfused vessel percentage in each group (n=5) **(H)** Quantification of extravasated dextran in each group (n=5)

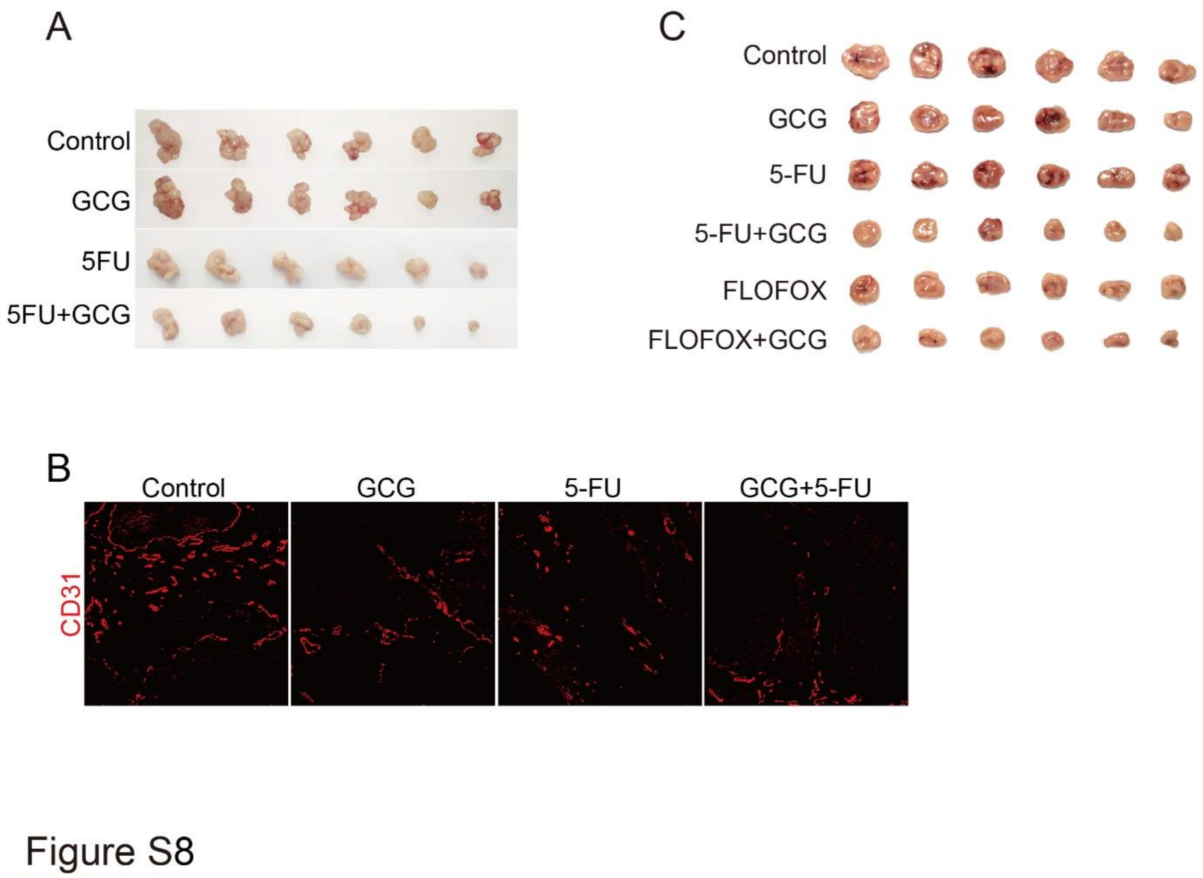

Figure S8. Glucagon enhanced chemotherapy in PDX and clinically relevant regimen (A) Tumor images of SW480 (n=6) **(B)** Blood vessel staining of PDX tumors (n=4) **(C)** Images of CT26 tumors treated with vehicle, GCG, 5-FU, FOLFOX, 5-FU+GCG, or FOLFOX + GCG. GCG, 20 μg/mouse; 5-FU, 12.5 mg/kg; Oxaliplatin, 1.5 mg/kg; Calcium Folinate, 4.5 mg/kg.

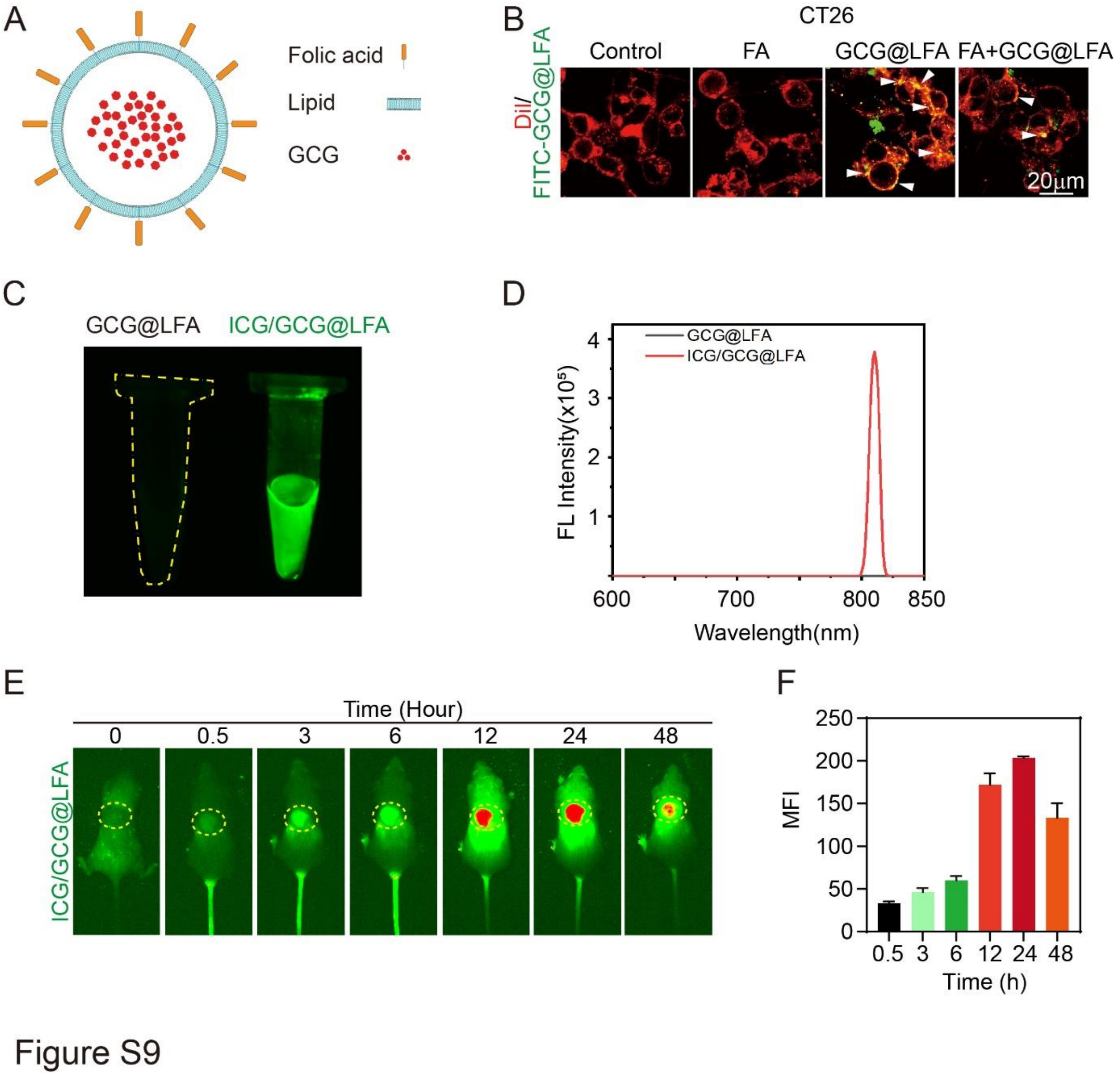

Figure S9. Synthesis and delivery of GCG@LFA *in vitro* and *in vivo* (A) Schematic presentation of GCG@LFA **(B)** Targeting cell membrane of GCG@LFA. FA, 1 mM; GCG@LFA 100 nM. **(C)** Photographs (from left to right, GCG@LFA, ICG/GCG@LFA, 0.4 mg/mL) taken under Azure Imaging Systems (c600, azure biosystems) **(D)** Fluorescence emission spectra, and corresponding fluorescence images of GCG@LFA and ICG/GCG@LFA (20 μg) with equivalent GCG concentration. λex =785 nm (n=3) **(E)** Real-time near-infrared imaging of mice bearing CT26 cell transplantation tumors after tail vein injection of ICG/GCG@LFA using a fluorescence imaging system (LSR-PS-II; Lasever Inc.) (n=3) **(F)** Semi-quantitation of intratumoral fluorescence changes in ICG/GCG@LFA with increase in post-injection time (n = 3).

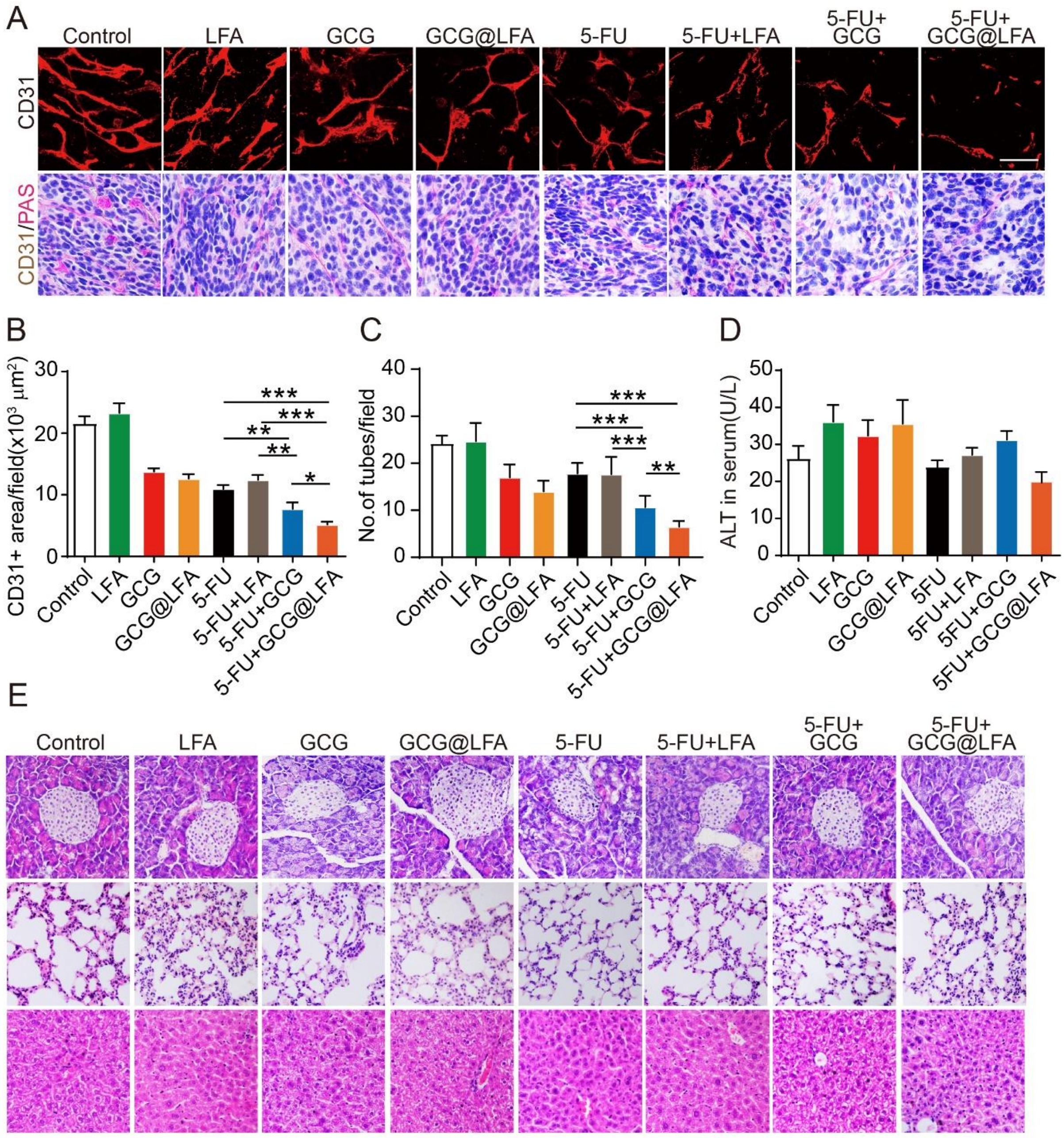

Figure S10

Figure S10. GCG@LFA enhanced 5-FU treatment without exhibiting toxicity (A) Blood vessel and pseudo-vessel staining in different treatment groups. bar=50 μm (n=5–8) **(B)** Quantification of $CD31^+$ vessel area (n=5–8) **(C)** Quantification of $PAS^+/CD31^-$ vessels in different treatment groups. Bar=100 μm (n=5) **(D)** Serum alanine aminotransferase (ALT) levels (n=5) **(E)** Hematoxylin and eosin staining of the pancreas, lungs, and liver from CT26 tumor-bearing mice under different treatment conditions (n=5)
